# Impact of the POPulation Medicine Multimorbidity Intervention in Xishui County (POPMIX) on people at high risk for COPD who smoke: protocol for the POPMIX-Smoking cluster randomised controlled trial

**DOI:** 10.1136/bmjopen-2025-112179

**Published:** 2026-05-06

**Authors:** Simiao Chen, Ke Huang, Zhoutao Zheng, Yuhao Liu, Shiyu Zhang, Wenjin Chen, Xingyao Tang, Zhong Cao, Lei Tang, Xunliang Tong, Jinghan Zhao, Liu He, Lirui Jiao, Yingping Wang, Tianying Zhao, Yingchi Luo, Qiande Lai, Xiangqin Lyu, Ruopeng Dou, Qiushi Chen, Aditi Bunker, Sebastian Vollmer, Pascal Geldsetzer, Dean Jamison, Till Bärnighausen, Ting Yang, Chen Wang, on behalf of the POPMIX study investigators, Chun Zhang

**Affiliations:** 1 School of Population Medicine and Public Health, Chinese Academy of Medical Sciences & Peking Union Medical College, Beijing, China; 2 Heidelberg Institute of Global Health, Faculty of Medicine and University Hospital, Heidelberg University, Heidelberg, Germany; 3 State Key Laboratory of Respiratory Health and Multimorbidity, Beijing, China; 4 Department of Pulmonary and Critical Care Medicine, China-Japan Friendship Hospital, Beijing, China; 5 National Center for Respiratory Medicine, Beijing, China; 6 School of Health Policy and Management, Chinese Academy of Medical Sciences & Peking Union Medical College, Beijing, China; 7 Guizhou Medical University, Guiyang, Guizhou, China; 8 Department of Pulmonary and Critical Care Medicine, National Center of Gerontology, Beijing Hospital, Chinese Academy of Medical Sciences Institute of Geriatric Medicine, Beijing, China; 9 Department of Health Policy and Management, The University of North Carolina at Chapel Hill Gillings School of Global Public Health, Chapel Hill, North Carolina, USA; 10 Center for Disease Control and Prevention of Xishui County, Zunyi, Guizhou, China; 11 Department of Pulmonary and Critical Care Medicine, People’s Hospital of Xishui County, Zunyi, Guizhou, China; 12 The Harold and Inge Marcus Department of Industrial and Manufacturing Engineering, Pennsylvania State University University Park, University Park, Pennsylvania, USA; 13 Department of Economics and Centre for Modern Indian Studies, University of Goettingen, Goettingen, Germany; 14 Division of Primary Care and Population Health, Stanford University Department of Medicine, Stanford, California, USA; 15 Chan Zuckerberg Biohub San Francisco, San Francisco, California, USA; 16 Department of Epidemiology and Biostatistics and Institute for Global Health Sciences, University of California, San Francisco, California, USA; 17 Department of Global and Population Health, Harvard University, Harvard T H Chan School of Public Health, Boston, Massachusetts, USA

**Keywords:** Multimorbidity, Primary Care, RESPIRATORY MEDICINE (see Thoracic Medicine), PUBLIC HEALTH

## Abstract

**Introduction:**

Tobacco use is a major contributor to the burden of chronic obstructive pulmonary disease (COPD) and other non-communicable diseases in China. People at high risk for COPD who smoke, particularly those with pre-existing chronic conditions, often remain underserved by conventional smoking cessation programmes. Population medicine offers a promising framework for proactively identifying high-burden diseases, managing multimorbidity and prioritising interventions for vulnerable populations.

**Methods and analysis:**

This protocol describes a stratified, two-arm cluster randomised controlled trial (Population Medicine Multimorbidity Intervention in Xishui County-Smoking) being conducted in Xishui County, a rural area of Guizhou Province, China. A total of 26 townships were stratified by population size and randomly assigned in a 1:1 ratio to receive either a multicomponent intervention or usual care. Eligible participants were individuals aged 35 years or older who smoked and were at high risk for COPD as identified by the COPD Screening Questionnaire. The intervention package integrates multiple components, including a digital smoking cessation programme, digital mental health support, community-based spirometry, tailored chronic disease management, health education and a performance-linked ‘pay-for-population’ scheme that aligns healthcare worker reimbursement with population health outcomes. Primary outcomes are smoking amount and nicotine dependence and secondary outcomes include COPD-related health outcomes, hypertension, diabetes, health risk behaviours, quality of life, healthcare utilisation and productivity loss. Follow-up occurs at 3, 6 and 12 months.

**Ethics and dissemination:**

Ethical approval has been granted by the Peking Union Medical College Ethics Committee (CAMS&PUMC-IEC-2024-042). Informed consent was obtained from all participants prior to enrolment. Results will be shared through peer-reviewed publication and (inter)national conference presentations.

**Trial registration number:**

NCT06458205.

**STRENGTHS AND LIMITATIONS OF THIS STUDY**

- Conducted in a rural, resource-limited Chinese setting, Population Medicine Multimorbidity Intervention in Xishui County-Smoking evaluates a novel, multicomponent population medicine intervention aimed at people who smoke and are at high risk for chronic obstructive pulmonary disease, integrating smoking cessation, mental health support and chronic disease management.
- The trial tests the implementation and effectiveness of a proactive, digitally-enabled modular-approach care model, shifting from a patient-centred, reactive approach to a population-centred, proactive one.
- The trial will generate evidence on the role of a ‘pay-for-population’ incentive mechanism in aligning primary care providers’ goals with public health outcomes, a crucial but understudied enabler for scalable population health strategies.
- The study addresses a critical evidence gap on managing multimorbidity and tobacco dependence concurrently, offering a potentially scalable framework for similar low-resource contexts globally.
- A limitation is that this is a very large, community-based, cluster randomised controlled trial within a mountainous terrain where WiFi is usually disconnected, making trial procedures challenging.

## Introduction

### Background and rationale

 The growing burden of chronic diseases and persistent health inequities has highlighted the need for healthcare approaches that extend beyond conventional, reactive and disease-centred models.^1^ Population medicine, an integrative discipline that combines clinical care with public health principles, has received increasing attention for its focus on proactive, population-level health improvement.^2^ Rather than treating individual conditions in isolation, it emphasises coordinated, comprehensive care that links individual behaviour with collective action to maximise long-term population health and equity. By prioritising conditions that are prevalent, preventable and amenable to broad intervention, this approach seeks to enable more efficient resource allocation and earlier, wider public health gains.

Tobacco use is a leading preventable risk factor for non-communicable diseases (NCDs) in China. Accounting for 40% of global cigarette consumption,^3^ smoking is responsible for approximately 20% of all deaths among middle-aged Chinese men and 3% among women.^4^,^6^ Tobacco-related NCDs, including chronic obstructive pulmonary disease (COPD), lung cancer and cardiovascular disease, account for 24% of all NCD deaths in China, significantly exceeding the global average of 15%.^7^ These conditions contribute approximately 1 year of a 3.5-year life expectancy gap attributable to seven major NCDs and injury-related priority conditions between China and the North Atlantic region.^8^ The economic burden of tobacco-related NCDs is profound, with projections indicating that between 2015 and 2030, these conditions will cost China 16.7 trillion yuan (US$2.3 trillion), equivalent to 0.9% of the nation’s annual income.^9^

Among tobacco-related NCDs, COPD is particularly burdensome in China,^10^ with a prevalence of 13.7% among adults over 40 years of age.^11^ China also faces the highest economic impact from COPD globally, with an estimated INT$1.36 trillion (95% uncertainty interval 1.03 to 1.80 trillion) in cumulative losses over 2020–2050.^12^ Smoking is the primary modifiable risk factor for COPD; 80%–90% of patients have a smoking history, and up to half of older people who smoke may develop the disease.^13 14^ Long-term and heavy smoking not only increases disease risk but also accelerates progression and elevates mortality.^15^ A meta-analysis covering 28 countries confirmed that COPD prevalence is significantly higher among people who currently smoke (15.4%) or formerly smoked (10.7%) compared with those who never smoked (4.3%).^16^ Smoking cessation can reduce COPD-related mortality, improve pulmonary function and decrease acute exacerbations.^17^ Yet smoking cessation support in China, particularly in rural primary care, remains inadequately integrated into routine services,^18^,^20^ and many providers lack the training, resources and incentives needed to deliver evidence-based cessation support.^21^

A core principle of population medicine is the early identification and proactive management of high-risk individuals before irreversible disease develops. In China, most patients with COPD are not diagnosed until advanced symptoms appear, with fewer than 1% of patients aware of their condition prior to symptom appearance and less than 6% having undergone spirometry.^22^ Many people at high risk for COPD who smoke already have respiratory symptoms, impaired quality of life and increased healthcare use despite lacking a formal diagnosis.^23^ They also commonly experience multimorbidity, including hypertension, diabetes and mental health disorders.^24 25^ In addition, smoking often clusters with other modifiable behavioural risks, including physical inactivity, unhealthy diet and harmful alcohol use, which contribute to COPD progression and other chronic diseases.^26^ These risks are interrelated and are poorly addressed by fragmented, single-disease models of care.^27 28^

Integrated management is therefore likely to be more appropriate than stand-alone smoking cessation support. Digital health may help extend such an approach in resource-limited settings. Digital smoking cessation interventions can provide scalable, tailored behavioural support and may overcome barriers related to geography, cost and workforce capacity.^29^,^32^ Studies indicate that digital cessation programmes not only improve smoking cessation rates but also support sustained abstinence, providing a cost-effective and adaptable solution for diverse populations.^33 34^ Despite evidence supporting the effectiveness of digital health interventions in promoting smoking cessation,^35 36^ their impact on patients with COPD remains insufficiently studied.

To advance population health, physicians are increasingly called on to extend their focus beyond individual clinical care and contribute to community-level health outcomes. Achieving this broader mandate, however, requires systemic support rather than an expansion of individual responsibilities in isolation. This protocol introduces a performance-linked ‘pay-for-population’ scheme that ties financial incentives for healthcare workers to concrete population-level indicators, including spirometry coverage, diagnosis rates, treatment initiation and reduction in acute exacerbations. By rewarding measurable improvements in population health outcomes, the pay-for-population mechanism aims to reorient primary care toward proactive, population-level management while allowing physicians to integrate population health activities into their existing roles in a sustainable manner.

Given the substantial burden of tobacco-related morbidity among high-risk individuals and current gaps in early detection and integrated care delivery, there is a critical need for scalable interventions that leverage digital tools and address multimorbidity in a systematic manner. This study aims to fill that gap by evaluating a population-level, multi-component intervention targeting people at high risk for COPD who smoke in a rural Chinese context. By integrating smoking cessation, mental health support, chronic disease management and digital health delivery into a unified care framework, this trial seeks to generate robust evidence for an innovative and context-sensitive model of preventive care that may inform future policy and practice in low-resource settings.

### Objectives

This study leverages a cluster-randomised controlled trial (cRCT) design to assess the effectiveness of a multimorbidity intervention package aimed at people at high risk for COPD who smoke in Xishui, China. Through this approach, we aim to investigate whether a multi-component multimorbidity intervention package affects the primary outcomes—amount of smoking and smoking dependence—and secondary outcomes, including COPD-related health outcomes, hypertension, diabetes, health risk behaviours, quality of life, healthcare utilisation and productivity loss.

## Methods and analysis

### Trial design

**Figure 1:**
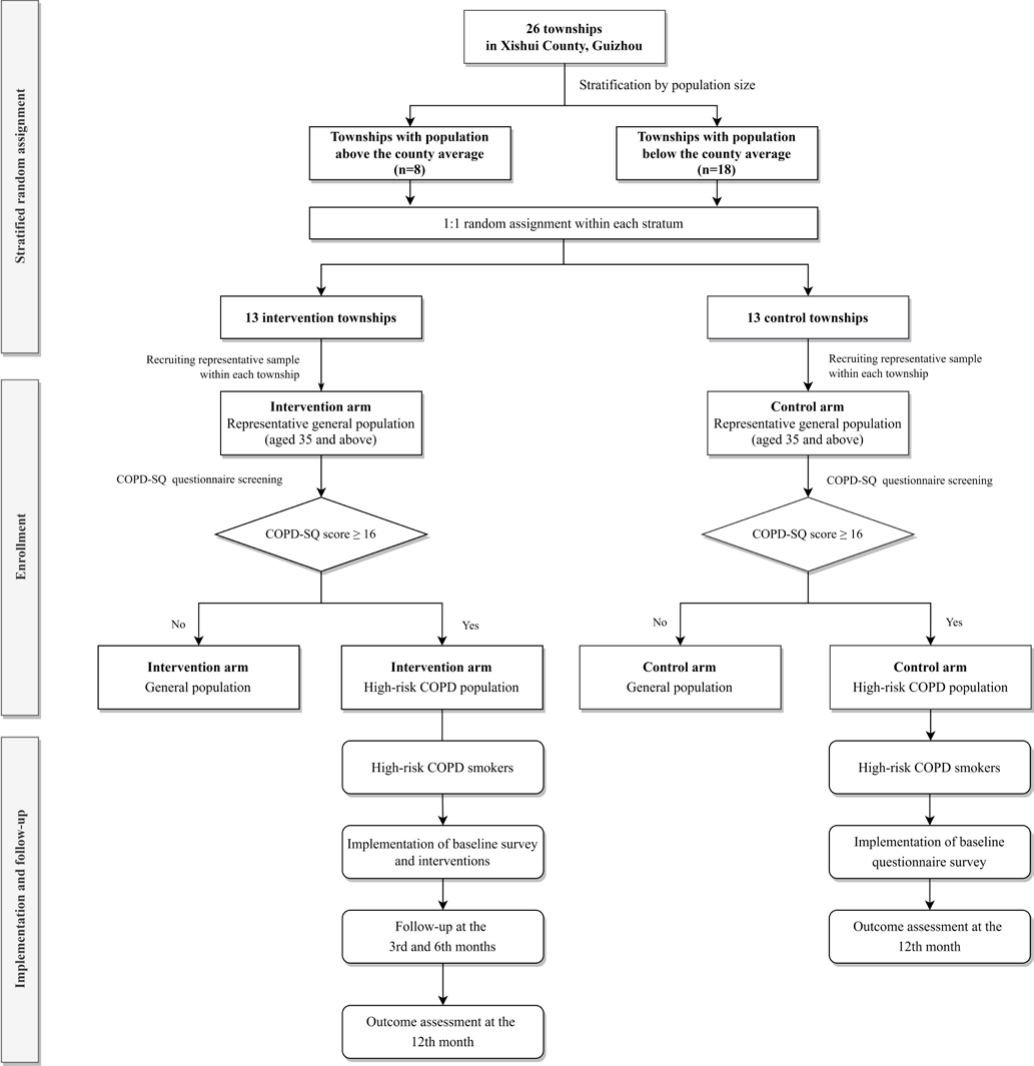
POPMIX-Smoking trial flow chart. COPD, chronic obstructive pulmonary disease; COPD-SQ, COPD Screening Questionnaire; POPMIX, Population Medicine Multimorbidity Intervention in Xishui County.

Population Medicine Multimorbidity Intervention in Xishui County-Smoking is a parallel, two-arm stratified cRCT conducted in Xishui, Guizhou (figure 1). The protocol was designed according to the guidance of the Standard Protocol Items: Recommendations for Interventional Trials (SPIRIT) 2025 Statement.^37^ The SPIRIT checklist for this protocol is provided in the online supplemental material (SPIRIT checklist). The trial is registered at ClinicalTrials.gov (NCT06458205).

Townships in Xishui were stratified based on whether their population size exceeds the average for the county to improve balance in community size-related characteristics. Each stratum was randomly allocated in a 1:1 ratio to either the intervention or control group using a computer-generated randomisation sequence. Participant recruitment was based on a comprehensive roster of permanent residents aged 35 years and above, provided by the local government as of 10 May 2024, with individuals selected from each township for enrolment. Community health workers and local officials initiated contact with potentially eligible residents using phone calls or home visits. During initial contact, residents were provided with a study information sheet, given the opportunity to ask questions, and invited to attend a community survey site for eligibility screening and baseline assessment if eligible. Informed consent was obtained from all participants prior to enrolment.

This study employed the COPD Screening Questionnaire (COPD-SQ), which was developed in 2013 by Zhou *et al*,^38^ to identify people at high risk for COPD. The COPD-SQ has a total score range of 0 to 38 and includes seven items: age, cumulative smoking history, body mass index (BMI), cough, breathlessness, family history of respiratory diseases and exposure to cooking-related smoke. Higher scores indicate greater risk. The instrument has been validated in multiple epidemiological studies and community-based screenings across China and is widely recommended by primary healthcare institutions as a reliable screening tool for COPD.^39^,^41^ Only participants with a COPD-SQ score of 16 or higher were included in this study.

### Setting

The study takes place across 26 townships within Xishui County, Guizhou Province. Located in the mountainous region of northern Guizhou, Xishui County is part of a province that ranks second nationally in smoking prevalence at 37.9%.^42^ Designated as a national experimental zone for comprehensive primary healthcare, Xishui County is characterised by innovative policy exploration as well as weak economic foundations (with its 2024 per capita gross domestic product (GDP) at only 52% of the national average) and limited human capital, making it a representative resource-constrained rural area. Xishui’s dual characteristics of economic underdevelopment and healthcare system experimentation provide a unique and appropriate setting to evaluate scalable multi-disease interventions tailored to populations facing resource scarcity.

### Trial participants (inclusion and exclusion criteria)

To be eligible for this trial, people at high risk for COPD who smoke must meet the following criteria: (1) be aged 35 years or older; (2) be a local resident who had stayed within a county township in the previous 3 months and would continue to stay within the same township for the next 12 months; (3) have a COPD-SQ score of 16 or higher; (4) provide written informed consent to participate in the study and (5) self-report current smoking or having quit smoking within the past 6 months. Participants are excluded if they meet any of the following conditions: (1) have a severe cognitive impairment that affects comprehension, decision-making or the ability to follow study procedures or (2) have complete loss of independent daily living ability, which may interfere with participation in assessments or adherence to the intervention.

### Intervention assignment

In this cRCT, a multicomponent smoking cessation intervention is implemented at the township level, employing a geographically stratified randomisation approach to ensure balance across communities with differing characteristics. A computer-generated randomisation sequence was used to allocate 13 townships to either the intervention or control group, with the allocation conducted by an independent statistician uninvolved in participant recruitment or intervention delivery. Given the nature of the intervention, this is an open-label trial. No formal blinding procedures were implemented. Healthcare providers are aware of cluster-level group allocation, and participants were aware of the care they received. To minimise expectation bias, participants are not informed of their specific group allocation, and explicit labels such as ‘intervention group’ are deliberately avoided. The control group receives only local usual care throughout the study period, with no additional intervention applied. Statisticians and investigators will be blinded to the group assignments wherever operationally feasible, consistent with best practices in the open-label population health and behavioural intervention trials.

To enhance adherence to the intervention, a structured follow-up protocol is used. Follow-up assessments are scheduled at 3, 6 and 12 months postbaseline. At the 3-month mark, participants receive telephone interviews, while in-person visits are conducted at the 6-month and 12- month follow-ups to reinforce engagement. During each follow-up, participants are provided with personalised feedback based on key health indicators such as spirometry results and smoking status, with comparative analyses against baseline data to highlight areas of improvement or concern. For participants who do not successfully quit smoking or who disengaged from the programme, the research team conducts barrier analyses incorporating baseline data to identify potential challenges (eg, low motivation or difficulties using digital tools). Adherence is systematically assessed through validated questionnaires, capturing indicators such as app usage frequency and the number of quit attempts. Additionally, automated reminders and tailored counselling are used to address issues of non-adherence. This dynamic follow-up framework aims to optimise the participant experience at each stage, providing continuous motivation and enabling adaptive strategy adjustments to support sustained engagement over the course of the trial.

### Interventions

**Figure 2:**
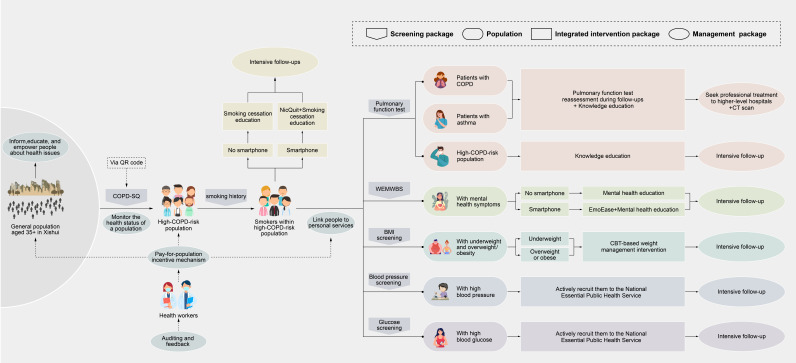
Integrated pathway of the multicomponent intervention package. BMI, body mass index; CBT, cognitive behavioural therapy; COPD, chronic obstructive pulmonary disease; COPD-SQ, COPD Screening Questionnaire; EmoEase, a CBT-based digital mental health intervention via WeChat; NicQuit, a CBT-based digital smoking cessation intervention via WeChat; QR, quick response; WEMWBS, Warwick–Edinburgh Mental Well-being Scale.

Participants assigned to the intervention group receive access to the intervention package, which was developed through iterative prototyping and stakeholder consultations. The specific eligibility criteria for each intervention are summarised in table 1. The strategies comprising the multicomponent intervention in this trial have been shown to be cost-effective and are endorsed by the *Lancet* Commission on Investing in Health.^8^ Figure 2 illustrates the overall structure and implementation pathway of the intervention package. Multicomponent interventions are implemented for people at high risk of COPD who smoke, including digital smoking cessation, mental health education, weight management, hypertension and diabetes care, pulmonary function testing and referral for treatment. Participants in the control arm received a pop-up notification on their mobile device immediately after completing the quick response (QR) code-based COPD-SQ screening. Those with high COPD risk were explicitly informed of their high-risk status, invited to complete the baseline face-to-face survey, and given a general recommendation to seek usual care at regular medical institutions independently. No further study-delivered intervention is provided. In Xishui, usual care largely follows a routine, reactive model, in which residents seek care on their own initiative rather than through proactive population management.

**Table 1:** Eligibility criteria for interventions

Population level	Target population	Intervention	Eligibility criteria
General population	General population	Health education	
	General population	Online screening with COPD screening questionnaire	Permanent residents aged 35 years and above
Trial participants	Individuals at high risk for COPD who smoke	Smoking cessation digital health interventions	Currently smoking or have quit within the last 6 months and own a smartphone.
	Individuals at high risk for COPD who smoke	Smoking cessation health education for individuals who smoke	Currently smoking or have quit within the last 6 months.
	Individuals at high risk for COPD who smoke	Community-based spirometry pulmonary function tests, interpretation of results and health education	Individuals with COPD-SQ ≥16
	Patients with COPD who smoke	Encouragement to seek professional medical treatment	Individuals with FEV1/FVC <0.7 postbronchodilation.
	Individuals who smoke and have mental health symptoms	Mental health digital health interventions	Individuals with Warwick Edinburgh Mental Wellbeing Scale (WEMWBS) <45 who own a smartphone
	Individuals who smoke and have mental health symptoms	Health education for people who smoke and have mental health needs	Individuals with WEMWBS <45
	Individuals who smoke and have hypertension	Hypertension management and education	Three consecutive measurements with average systolic blood pressure ≥140 mmHg and/or diastolic blood pressure ≥90 mmHg.
	Individuals who smoke and have diabetes	Diabetes management and education	Fasting blood glucose ≥7.0 mmol/L or random blood glucose ≥11.1 mmol/L.
	Individuals who smoke and have underweight, overweight or obesity	Weight management interventions	Individuals with BMI <18.5 or BMI ≥24.0.
Health providers	Health providers	Intrinsic incentive mechanism	Primary care providers who engage with the investigation and intervention
	Health providers	Extrinsic incentive mechanism	Primary care providers who engage with the investigation and intervention

BMI, body mass index; COPD, chronic obstructive pulmonary disease; COPD-SQ, COPD Screening Questionnaire; FEV1, forced expiratory volume in 1 s; FVC, forced vital capacity.

**Figure 3:**
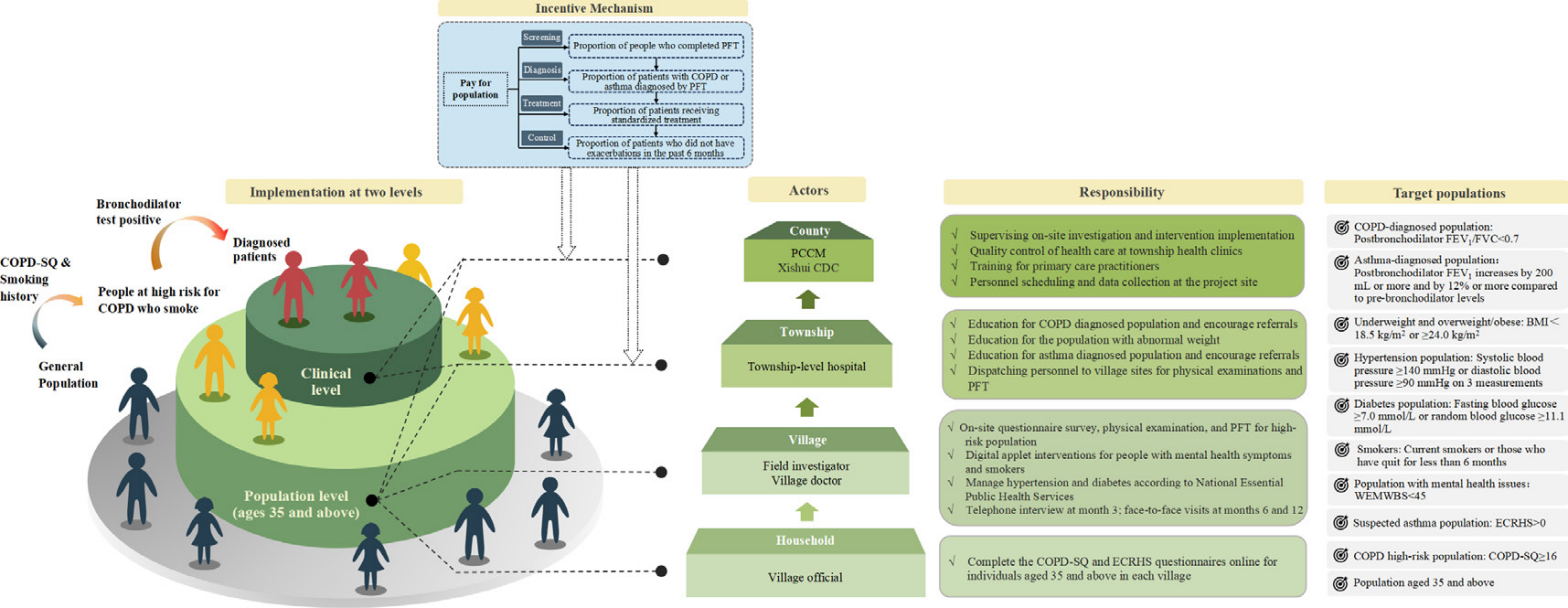
Implementation structure and execution mechanism of the multicomponent intervention package. BMI, body mass index; CDC, Centre for Disease Control and Prevention; COPD, chronic obstructive pulmonary disease; COPD-SQ, COPD Screening Questionnaire; ECRHS, European Community Respiratory Health Survey; FEV₁, forced expiratory volume in 1 s; FVC, forced vital capacity; PCCM, Pulmonary and Critical Care Medicine; PFT, pulmonary function test; WEMWBS, Warwick–Edinburgh Mental Well-being Scale.

The implementation of the multicomponent intervention package is supported by a multilevel delivery structure and performance-linked incentive mechanism. Figure 3 illustrates the organisational structure, population stratification, incentive design and responsibility distribution across administrative levels (county, township, village, household) for delivering the intervention in Xishui. This figure also specifies the eligibility criteria for each target subpopulation, clarifying how risk stratification and service delivery were operationalised in the field.

The multicomponent intervention package comprises the following components:

#### Health education

All representative permanent residents in the intervention townships receive general health education. This education focuses on increasing awareness of chronic disease prevention (eg, COPD, asthma, diabetes and hypertension), mental health and healthy behaviours (eg, tobacco cessation, physical activity and healthy diet). Health workers distribute printed educational materials during household visits and community events, share videos and health messages through intervention-arm WeChat Group, and conduct brief verbal sessions to inform and empower community members about relevant health issues.

#### Online screening for COPD

All representative permanent residents aged 35 years and above are invited to complete an online screening questionnaire targeting symptoms of COPD. The digital form, accessed via QR code, includes the validated COPD-SQ. Residents who screen positive (COPD-SQ≥16) are identified as high-COPD-risk individuals and referred for further interventions.

#### Smoking cessation digital health interventions

NicQuit is a WeChat-based digital smoking cessation intervention designed for individuals who are currently smoking or have quit within the past 6 months. The programme integrates structured, interactive modules grounded in cognitive-behavioural therapy (CBT), acceptance and commitment therapy and other evidence-based psychological frameworks to support behaviour change. Core content includes strategies for managing smoking triggers, cognitive restructuring techniques, behavioural activation exercises and reinforcement methods to support sustained abstinence. Modules are delivered through simulated conversational dialogues, enabling participants to engage with the material in an interactive and intuitive format. The intervention is specifically targeted at individuals familiar with smartphone technology, ensuring accessibility and usability. Personalised notifications and reminders are delivered through the WeChat platform, encouraging participants to regularly engage with the cessation plan and maintain adherence.

#### Smoking cessation health education for people who smoke

Participants in the intervention group receive targeted health education to reinforce the importance of smoking cessation. This education focuses on the health risks associated with smoking and the benefits of quitting, providing participants with evidence-based information and practical advice. Health education is delivered by primary healthcare providers through brief verbal counselling, printed posters and videos or messages shared through WeChat groups.

#### Community-based spirometry, interpretation of results and health education

High-COPD-risk individuals in the intervention group receive real-time pop-up alerts directing them to a community gathering place for spirometry testing, which is conducted using BH-AX-MAPG spirometry equipment. Those who screen positive for COPD are referred to the county hospital for CT and a formal diagnosis. In addition, they receive health education on the risks of COPD and how to prevent and manage the disease, delivered through verbal communication by primary healthcare providers and supplemented with printed materials for distribution.

#### Mental health digital health interventions

A CBT-based digital mental health intervention, EmoEase,^43^ is offered to individuals experiencing mental health symptoms (Warwick-Edinburgh Mental Well-being Scale, WEMWBS <45) who have a smartphone. This WeChat-based programme includes psychoeducation, mood tracking, guided CBT exercises and self-regulation techniques. The programme emphasises the link between mental health and respiratory symptoms, aiming to enhance coping, treatment adherence and sustained behaviour change.

#### Health education to people who smoke with mental health symptoms

Individuals who smoke and have co-occurring mental health symptoms are offered specialised health education tailored to the challenges they face. This health education includes guidance on how to manage mental health symptoms and is delivered through verbal communication, videos/messages via WeChat Groups and supplemented with printed materials for distribution.

#### Encouragement to seek professional medical treatment at higher-level hospitals for spirometry-defined patients with COPD and asthma

Participants diagnosed with COPD or asthma through spirometry—defined as those with a post-bronchodilator forced expiratory volume in 1 s (FEV1)/forced vital capacity (FVC) ratio of <0.7 for COPD and those with a ≥200 mL and ≥12% improvement in FEV1 post-bronchodilation for asthma—are encouraged to seek professional medical treatment at higher-level hospitals for further diagnosis and management.

#### Hypertension and diabetes management

The goal of this intervention is to actively enrol individuals who smoke and have elevated blood pressure (≥140/90 mmHg)^44^ or elevated blood glucose (random blood glucose ≥11.1 mmol/L or fasting blood glucose ≥7.0 mmol/L) into the National Essential Public Health Service in China.^45^ These participants are also provided health education on hypertension and diabetes through verbal counselling by trained community health workers or general practitioners and printed materials for distribution.

#### Weight management interventions

Participants with a BMI <18.5 (underweight) or ≥24.0 (overweight or obese) receive structured weight management counselling at baseline and at each follow-up visit. Each session lasts approximately 5 to 10 min. During these sessions, healthcare providers use motivational interviewing techniques supported by CBT-informed prompts to help participants identify weight-related barriers, reflect on the perceived advantages and disadvantages of behavioural change, and set realistic, actionable goals. Counselling includes tailored advice on diet, physical activity and daily routines to support healthy weight gain for underweight participants and gradual, safe weight loss for those with overweight or obesity. To reinforce key messages, participants are also provided with printed educational materials to take home and with videos/messages via WeChat Groups.

#### Pay-for-population mechanism

A comprehensive incentive strategy, integrating both extrinsic and intrinsic components, has been introduced to motivate primary care providers to actively participate in population health interventions. Extrinsic motivation is provided through results-based financial rewards aligned with four key stages of care: screening, diagnosis, treatment and control. At the township level, health providers are assessed using four performance indicators: the proportion of residents aged 35 years and above completing pulmonary function testing, the proportion of high-risk individuals identified in the initial screening (defined as COPD-SQ ≥16) subsequently diagnosed with COPD, the proportion of confirmed patients with COPD receiving standardised inhaled treatment, and the proportion of patients who have not experienced acute exacerbations in the past 6 months. The county hospital’s respiratory department and the county Centre for Disease Control and Prevention are evaluated using the same set of indicators, with data aggregated across all 13 intervention townships. In addition to offering providers an extrinsic, results-based financial incentive, they are also offered specialised training and capacity-building opportunities in an effort to appeal to their intrinsic motivation, support proactive service delivery and foster a strong sense of responsibility for population health.

### Outcomes

The primary outcomes of this study focus on assessing changes in smoking behaviour and dependence over the course of the intervention. The first primary outcome is the participant’s amount of smoking, measured by the self-reported average number of cigarettes smoked per day at baseline and at the 3, 6 and 12-month follow-ups to capture changes in smoking intensity. The second primary outcome is smoking dependence, assessed using the Chinese version of the Fagerström Test for Nicotine Dependence (FTND),^46 47^ which scores nicotine dependence on a scale from 0 to 10, with higher scores indicating greater dependence. The Heaviness of Smoking Index (HSI),^48^ which ranges from 0 to 6, is used to supplement the FTND by further quantifying the severity of nicotine addiction. The FTND and HSI were assessed at baseline and are reassessed at the six- and 12 month follow-ups. Secondary outcomes examine broader health and behavioural changes, providing insights into the intervention’s wider impact. Key measures include self-rated health status using the EuroQol 5-Dimension 5-Level (EQ-5D-5L) scale, a validated tool that translates responses into a health utility value between 0 (death) and 1 (perfect health).^49 50^ Other secondary outcomes focus on chronic condition management, including the number of conditions effectively controlled (such as COPD, hypertension and type 2 diabetes), lung function (measured by FEV_1_ through spirometry), and treatment adherence to prescribed COPD management plans. Additionally, lifestyle habits such as physical activity, diet and alcohol consumption are monitored to capture changes in health behaviours associated with the intervention (see table 2 for primary outcomes and online supplemental table S1 for secondary outcomes).

**Table 2:** Primary outcomes of the cRCT

Primary outcomes	
Amount of smoking	Definition: Average number of cigarettes smoked per day Functional form: Continuous Measurement: Self-reported response
Smoking dependence	Definition: A scale that measures the degree of smoking dependence Functional form: Continuous Measurement: Score on the Chinese version of the Fagerström Test for Nicotine Dependence,^46 47^ which ranges from 0 to 10 with higher scores representing more severe nicotine dependence; additionally measured by score on the Heaviness of Smoking Index,^48^ which ranges from 0 to 6 with higher scores representing worse nicotine dependence

cRCT, cluster randomised controlled trial.

### Timeline

This study was formally launched in June 2024, with participant recruitment conducted across the entirety of Xishui County. A longitudinal follow-up design was adopted, in which participants assigned to the intervention group are required to complete four standardised assessments: a baseline assessment (conducted on-site), a 3-month follow-up (via telephone), a 6-month reassessment (on-site) and a final 12-month evaluation (on-site). All data collection is expected to be completed by 31 March 2026. Analyses will be conducted after the dataset has been finalised and locked.

A stratified spirometry testing protocol was developed based on participants’ clinical characteristics. Individuals identified as at high risk for COPD underwent prebronchodilator pulmonary function tests at the time of baseline enrolment and were scheduled to complete the identical pre-bronchodilator assessment at the 12-month follow-up. Postbronchodilator spirometry was performed in participants with a confirmed COPD diagnosis, or a prebronchodilator FEV_1_/FVC ratio <70%, at the baseline, 6-month and 12-month follow-up. All assessments were performed by uniformly trained providers to ensure consistency and accuracy in data collection.

The 3-month follow-up consists of a structured telephone interview aimed at monitoring adherence to the intervention and tracking changes in health status. Comprehensive on-site reassessments at 6 and 12 months replicate the baseline evaluation procedures, including standardised questionnaires, clinical examinations and pulmonary function tests. In contrast, the control group follows a simplified protocol, comprising only the baseline questionnaire and a final on-site assessment at 12 months. A detailed schedule of data collection activities for each study phase is provided in online supplemental table S2.

### Sample size and power calculation

The sample size was determined to detect a mean reduction of three cigarettes per day in the intervention group compared with the control group, with a standard deviation of six cigarettes per day, consistent with estimates from prior trials of digital smoking cessation interventions in Chinese populations.^51 52^ Sample size calculation followed the formula for population-based stratified cRCTs proposed by Crespi.^53^ The intraclass correlation coefficient (ICC) was assumed to be 0.05, a conservative estimate based on previous community-based trials.^54^ Based on population data from the 26 townships, the expected cluster size was estimated to be 308. With 13 clusters per arm, the formula yielded a required sample size of 1374 participants per arm, or 2748 in total. To account for an anticipated 20% loss to follow-up, the required number of participants to recruit was inflated to 3435. Due to budgetary and operational considerations in this pragmatic trial, we set a feasible recruitment target of 7400 high-COPD-risk participants. Given the estimated 25% prevalence of high COPD risk among adults aged ≥35 years,^55^ screening approximately 44 000 individuals was planned to identify the required number of eligible participants.

We conducted sensitivity analyses to calculate the minimal detectable differences in amount of smoking across a range of plausible ICC values, with 13 clusters per arm, 80% statistical power and a two-sided significance level of 0.025. The results of these sensitivity analyses are presented in table 3, confirming that our final sample size is sufficient to detect clinically meaningful between-group differences across a range of realistic ICC assumptions.

**Table 3:** Minimal detectable difference in amount of smoking

Proposed sample size	ICC=0.005	ICC=0.01	ICC=0.025	ICC=0.05
2500	1.38	1.75	2.56	3.52
3000	1.26	1.60	2.34	3.21
3500	1.17	1.48	2.16	2.97
4000	1.09	1.39	1.98	2.74

ICC, intraclass correlation coefficient.

### Data collection plan

To comprehensively evaluate the effectiveness of the multi-component intervention (table 4), a multidimensional data collection framework has been established. At the initiation of the study, all enrolled participants were required to complete structured questionnaires covering demographic characteristics, smoking history, nicotine dependence (assessed using the FTND), mental health status (measured via Patient Health Questionnaire-9 (PHQ-9) for depression, Generalised Anxiety Disorder-7 (GAD-7) for anxiety and the WEMWBS for well-being), daily health practices and history of chronic diseases. In parallel, participants underwent comprehensive physical examinations to obtain measurements of height, weight, BMI, waist circumference, heart rate, blood pressure and blood glucose levels. Respiratory function was assessed using spirometry to evaluate pulmonary health and detect airflow limitation. For people at high risk of COPD who smoke in the intervention arm, prebronchodilator pulmonary function tests were conducted at baseline and are again conducted at 12 months to longitudinally monitor changes in lung function. In participants with a confirmed diagnosis of COPD in the intervention arm, additional spirometry assessments—both prebronchodilator and postbronchodilator—were performed at baseline and 12 months to enable comparative evaluation with an additional prebronchodilator assessment at 6 months. Follow-up assessments are conducted at three key time points: 3 months (via telephone), 6 months (on-site) and 12 months (on-site).

**Table 4:** Characteristics of Xishui County, Guizhou

	Xishui County
Total population size (*10 000)	30.20*
Number of rural townships	22
Number of villages within rural townships	210
Average rural township size (# of people)	9518
Number of urban townships	4
Number of communities within urban townships	47
Average urban township size (# of people)	23 150

* Total population size listed in table 4 was obtained from the Xishui authority. The figures here reflect permanent residents as of 10 May 2024 and are limited to individuals who had already stayed in Xishui County for 3 months in 2024 and would continue to stay within the same township for the next year.

At each follow-up, core indicators from the baseline assessment are re-evaluated, including self-reported smoking status (eg, daily cigarette consumption and dependence level), COPD management outcomes (respiratory function via spirometry, frequency of acute exacerbations and treatment adherence), and chronic disease-related metrics (blood pressure, blood glucose and BMI). Mental health status is reassessed using the same instruments (PHQ-9, GAD-7, WEMWBS) and health-related quality of life is measured using the EQ-5D-5L scale. Lifestyle factors—including physical activity, alcohol consumption and dietary habits (with attention to sugar, preserved food and vegetable intake)—are continuously monitored through self-reported data. Utilisation of healthcare services, including outpatient visits and hospital admissions, is also recorded. To ensure high data quality, all information is collected by professionally trained field staff following standardised procedures and entered into an electronic data capture (EDC) system equipped with built-in validation mechanisms to minimise entry errors and enhance data reliability.

### Data management

Study data will be collected and managed using a web-based EDC system (einMatrix). All data are entered electronically in real time and are subject to automated validation to ensure accuracy and completeness. Trained field investigators use mobile terminal devices to complete standardised electronic case report forms, with collected information immediately transmitted to a centralised database. The platform incorporates multiple built-in data quality control modules, including range checks, logical consistency validation and missing data alerts, thereby significantly enhancing data accuracy. Any outlier values flagged by the system are subject to manual verification by a dedicated data review team. Electronic forms follow a unified design format with precoded response options, ensuring comprehensive documentation of participants’ demographic, clinical and behavioural data.

Physiological parameters such as pulmonary function, blood pressure, BMI and blood glucose concentration are directly entered into the system by trained operators. Notably, spirometry data are transmitted from specialised devices and subjected to automated quality assessment procedures to ensure reliability. All study data are stored in a de-identified format within an encrypted database, with strict safeguards in place to protect participant privacy. The database is governed by a tiered access control system, with access granted only to core personnel such as the principal investigator, collaborating researchers and data analysts, ensuring secure and compliant data use.

### Statistical analysis

In accordance with the intention-to-treat principle, data from all randomised participants will be included in the final analysis. The primary outcomes will be analysed using a generalised linear mixed-effects model. Appropriate link functions and error distributions will be selected based on outcome type (eg, identity link with Gaussian distribution for continuous outcomes; logit link with binomial distribution for binary outcomes). We will report both unadjusted estimates and estimates adjusted for prespecified covariates, including age, sex, education level, marital status, baseline outcome measures and relevant comorbid conditions. Baseline characteristics of both arms will be presented to assess postrandomisation balance and inform covariate adjustment decisions. To address the risk of type I error inflation due to multiple testing, distinct adjustment strategies will be applied. The Bonferroni correction will be used for primary outcomes, while the Benjamini–Hochberg procedure will be applied to control the false discovery rate at the 5% level for all secondary outcomes.

To explore potential mechanisms and component effects of the integrated intervention, we have prespecified several complementary analyses. First, regression discontinuity designs (RDDs) will be employed to estimate the local average treatment effects of specific intervention components triggered by clinically defined thresholds. Second, to elucidate how the intervention package reshapes behaviours and experiences among target populations and healthcare providers, we will conduct a series of qualitative studies. Semistructured interviews will be carried out with high-COPD-risk participants, primary care physicians, community health workers and policymakers in the intervention arm. These qualitative investigations will complement the main trial findings by providing insights into the mechanisms of action, implementation processes and subjective experiences of receiving care under the population medicine framework.

### Monitoring

To ensure scientific rigour, independence and participant safety, we appointed an independent Data and Safety Monitoring Board (DSMB) comprising experts in public health, epidemiology, clinical research and respiratory disease. An external advisory committee provided additional oversight and international perspectives. The DSMB convened three meetings to review descriptive summaries from baseline and follow-up assessments in the intervention arm for process monitoring, including adherence to intervention components, implementation logistics and data completeness and quality. A formal interim analysis was pre-specified and conducted in February 2026. At this analysis, the biostatistics team presented emerging effect estimates comparing the intervention and control arms on the primary outcomes to the DSMB. By this time, the trial was nearing completion, as field workers confirmed that the majority of eligible participants had been contacted.

Stopping guidance was prespecified for safety, efficacy, futility and feasibility:

1. Any death or serious adverse event will be reported to the DSMB, which will assess relatedness to the intervention and advise whether the trial should be paused or stopped.
2. Null findings, suggesting that the intervention had no detectable effect and indicating that the study should be terminated.
3. Statistically significant findings, indicating that the study had achieved its primary endpoint and should be concluded.
4. If a positive trend is observed but statistical significance is not reached, the trial should continue as planned to determine the robustness of the effect. However, the study would be terminated if field workers confirmed that all eligible participants had been contacted and no additional individuals remained to undergo follow-up assessments.

### Patient and public involvement

None.

## Ethics and dissemination

### Research ethics approval

Ethics approval has been obtained from the Peking Union Medical College Ethics Committee (approval number: CAMS&PUMC-IEC-2024-042). A continuing ethics review was completed in June 2025, and updated approval was granted under approval number CAMS&PUMC-IEC-2025-063. To protect participant privacy, all identifying information will be removed from the dataset, and data will be anonymised before analysis. Participants have the right to withdraw from the study at any point, with no consequences for their standard healthcare access. The study is committed to upholding the highest standards of ethical research conduct, ensuring that all participants are treated respectfully and that their health and personal information are safeguarded throughout the research process. All procedures are carried out in accordance with relevant national and international guidelines and regulations, including the Declaration of Helsinki.

### Protocol amendments

The Ethics Commission of the Medical Faculty of Peking Union Medical College was contacted and notified about any protocol amendments requiring their acceptance. Following the approval of the Ethics Committee and before implementation, all research members were informed about protocol amendments.

In this trial, an unavoidable amendment was made to the primary outcome measures during the trial planning and preparation phase, before participant enrolment began. Although outcome modifications are generally discouraged, the change was necessitated by a non-negotiable regulatory restriction. Specifically, we initially planned to measure carbon monoxide (CO) levels as an objective biomarker of smoking status. However, due to government regulations prohibiting the purchase of fixed assets, including CO monitoring devices, we were unable to acquire the necessary equipment. As a result, CO measurement was removed from the primary outcomes, and adjustments were made accordingly. In addition, healthcare utilisation data (outpatient/inpatient visits and medical expenditure) originally planned for extraction from electronic health records became inaccessible due to unexpected government regulations; these outcomes are now measured through self-report in study surveys. These adjustments are reflected in the updated protocol. This amendment was approved by the Ethics Committee, and all research personnel were notified before implementation to ensure consistency in data collection and analysis.

### Consent and withdrawal

Participation in this study required the provision of written informed consent (see online supplemental material (informed consent form). We provided written study information and the informed consent form to eligible individuals. A study manager explained the study aims, procedures, potential risks and benefits, and answered questions, allowing sufficient time for decision-making. A witness was present when required, specifically when a participant was unable to read the consent materials (eg, illiteracy/limited literacy) or otherwise could not review the form independently. In these cases, the consent form was read aloud, the participant indicated consent by thumbprint, and the witness co-signed to attest that the information was accurately conveyed and that consent was given voluntarily before enrolment.

A decision not to participate in this study will not bear any further consequences for the individual. Every participant is free to refuse or discontinue data collection at any stage. Importantly, participation does not require relinquishing any concomitant care. While there are no special criteria for modifying or discontinuing allocated interventions, we will document and report reasons for attrition in future publications.

### Confidentiality

Throughout the study, all data have been handled in accordance with strict confidentiality protocols, and data collection has conformed to requirements of the national legislation on data protection. Digital data are stored in password-protected files on secure servers at the Chinese Academy of Medical Sciences, with access restricted to authorised research team members only. Any data shared with third parties for research purposes is anonymised prior to transfer. Informed consent forms and other participant-related documents are securely stored during the study and will be retained in locked facilities at the Co-Principal Investigator’s office premises thereafter.

### Dissemination

Study findings will be disseminated through publication in peer-reviewed scientific journals and presentation at national and international academic conferences. Key results will also be communicated to local health authorities and participating healthcare institutions to support translation into practice.

## Discussion

This protocol describes a cRCT designed to evaluate the effectiveness of a population medicine multimorbidity intervention for people at high risk of COPD who smoke in Xishui, China. The intervention aims to address both the physical and mental health needs of this vulnerable population by integrating multimorbidity management into routine care.

Evidence suggests people who smoke and have COPD face unique challenges in quitting, including longer smoking histories, lower self-efficacy and greater psychological dependence.^56^ These factors, compounded by frequent multimorbidity involving hypertension, diabetes, depression and anxiety, create complex clinical needs that vertical disease-specific programmes are ill equipped to address, particularly in rural communities where care is fragmented and specialist access is limited. Our intervention therefore adopts a unified, person-centred framework that integrates screening, follow-up and multimorbidity management rather than relying solely on reactive clinic visits.

A key feature of this intervention is its alignment with a broader shift in healthcare—from treating isolated illnesses in individual patients to managing health holistically at the population level. This evolving perspective calls for the early identification of high-burden conditions and a focused effort on those most at risk, such as individuals living with chronic diseases in rural areas. Realising this vision also depends on enabling physicians to act not only as caregivers to individuals, but also as stewards of community health—supported by systems like pay-for-population that align provider incentives with measurable improvements in population health.

The integrated nature of this intervention, which combines behavioural, medical and digital health strategies, holds promise for addressing the multifaceted needs of people at high risk of COPD who smoke. Smoking cessation remains one of the most effective strategies for reducing COPD-related mortality and morbidity.^57^ By offering both digital interventions like NicQuit and EmoEase alongside in-person health education and support, this study aligns with emerging trends in using mobile health technologies to improve health outcomes in resource-limited settings. The adoption of digital tools has been shown to be an effective method for increasing accessibility to smoking cessation resources, especially in rural communities where traditional healthcare infrastructure may be limited.

One of the major strengths of this study is its comprehensive, community-based intervention, which combines medical management with health behaviour change strategies to target both the underlying causes and the clinical manifestations of COPD. The use of a cRCT design allows for robust evaluation of the intervention’s impact at the population level, with the potential to inform public health strategies in rural areas of China. This is particularly important in regions like Xishui, where healthcare access may be limited and where public health interventions targeting high-risk populations are essential for improving outcomes. Furthermore, this study addresses a significant gap in the existing literature, as interventions for people at high risk of COPD who smoke in China remain scarce. The integration of a multimorbidity intervention could lead to more sustainable improvements in health outcomes, not just for COPD, but also for related comorbidities that disproportionately affect people who smoke.

However, there are potential challenges in both implementing the intervention and evaluating its effectiveness. First, the intervention’s success depends on participant engagement, particularly among older populations who may struggle with technology or behavioural change. The digital components of the intervention, if not adequately tailored, may face resistance or low uptake, especially in rural settings. Furthermore, the study’s results may be influenced by local contextual factors such as healthcare infrastructure and socioeconomic status, which could limit the generalisability of the findings beyond Xishui. Additionally, while the study’s design is rigorous, it is important to acknowledge that achieving high compliance rates for both the intervention and follow-up assessments could be challenging, especially in a community setting. Addressing barriers to adherence will be crucial for ensuring that the study’s findings are meaningful and reliable. Finally, the multi-faceted nature of the intervention, although reflective of real-world integrated care delivery, inherently limits our ability to isolate the individual contribution of each component to the overall treatment effect. This intervention was deliberately designed as a synergistic package in close collaboration with local policymakers, who prioritise overall effectiveness over component-level decomposition. Nevertheless, to partially address this interpretive complexity, we have prespecified exploratory analyses such as RDDs for threshold-triggered components.

This protocol outlines a promising intervention aimed at reducing the burden of COPD and tobacco-related diseases in China, particularly among people at high risk for COPD who smoke in Xishui. This study is timely, as it directly supports China’s national public health strategy by focusing on the prevention and management of COPD, which was integrated into the National Essential Public Health Service in 2024.^58^ By targeting multiple health outcomes through a multicomponent, community-level intervention, this study offers a potentially scalable model for addressing the needs of individuals with COPD and related co-occurring conditions. The findings from this trial could have significant implications for public health policy and the management of chronic diseases in underserved populations, especially in rural areas. The intervention could provide evidence for incorporating similar multimorbidity management strategies into routine care for patients with COPD in China and beyond. This study also has the potential to inform broader efforts to reduce smoking rates and improve mental health outcomes in high-risk populations, ultimately contributing to the reduction of the substantial health and economic burden caused by tobacco-related diseases.

If the intervention is shown to be effective, future research should evaluate its transferability to other regions and healthcare contexts and assess its longer-term scalability and sustainability. Additionally, further evaluation of the cost-effectiveness of this intervention will be essential for determining its scalability and sustainability in resource-limited areas. Ultimately, this study represents an important step towards improving the health outcomes of people at high risk for COPD who smoke and contributing to the ongoing fight against tobacco-related diseases in China.

Building on these findings, we hope this study will catalyse a paradigm shift in the role of primary and community health workers. In conventional health systems, frontline providers often operate passively, waiting for patients to present at clinics. This trial introduces mechanisms such as pay-for-population, performance-linked process metrics and home-based outreach to actively engage providers in population screening, health promotion and continuous care. This transformation—from a patient-centred, reactive model to a proactive, population-centred approach—is at the heart of population medicine. We believe this shift has the potential to reshape public health governance not only in China but also in other low-income and middle-income countries striving for equitable, sustainable health system reform.

In summary, this protocol presents a multicomponent intervention for people at high risk of COPD who smoke, combining smoking cessation, COPD management, mental health support and weight and chronic disease management. Using a cRCT design, the study aims to provide robust evidence on the effectiveness of this multimorbidity intervention in rural China. If successful, it could serve as a model for future population medicine strategies and inform policy decisions for managing COPD and tobacco-related diseases. Further research should explore the cost-effectiveness and scalability of this intervention to determine its long-term impact and broader applicability across different healthcare contexts.

## Supplementary material

10.1136/bmjopen-2025-112179 online supplemental file 1

10.1136/bmjopen-2025-112179 online supplemental file 2

10.1136/bmjopen-2025-112179 online supplemental file 3
