# Supplementary material for: Impact of the POPulation Medicine Multimorbidity Intervention in Xishui County (POPMIX) on people at high risk for COPD who smoke: protocol for the POPMIX-Smoking cluster randomised controlled trial
Source: BMJ Open. 2026 May 6;16(5):e112179. doi: 10.1136/bmjopen-2025-112179 (PMC13150926; doi:10.1136/bmjopen-2025-112179)
Supplement: online supplemental file 2 [file bmjopen-16-5-s002.docx]

**Supplementary Table S1. Secondary outcomes**

| **Secondary outcomes** |  |
| --- | --- |
| **Quality of life indicators** | |
| **Self-rated health status** | Definition: General self-assessed health status  Functional form: Continuous  Measurement: EQ-5D scale, ranging from 0 to 1 continuously; 0 represents death, and 1 represents perfect health^1^ |
| **Number of chronic conditions controlled** | Definition: The number of conditions controlled among seven objectively measured chronic health conditions (COPD, asthma, depression symptoms, anxiety symptoms, BMI, hypertension, and type 2 diabetes)  Functional form: Counting data  Measurement: Through objective physical examination or a validated scale |
| **Smoking-related indicators** | |
| **Physical proof of cigarettes** | Definition: Response to the question “Can you show me your cigarettes?”  Functional form: Binary  Measurement: Self-reported response and visible proof of cigarette possession to study field workers |
| **Knowledge and awareness of COPD** | |
| **Self-awareness of COPD** | Definition: Response to the question “Have you ever been diagnosed with COPD?”  Functional form: Binary  Measurement: Self-reported response |
| **COPD knowledge** | Definition: Responses to excerpted questions of the COPD Knowledge Questionnaire; selected questions ask about information the general population should know about COPD Functional form: Continuous  Measurement: Self-reported responses to questions; scores range from 0 to 7 with higher scores indicating a greater number of correct statements chosen by the respondent^2^ |
| **Care cascade indicators for COPD** | |
| **COPD screening** | Definition: Response to the question “Have you ever had a pulmonary function test?”  Functional form: Binary  Measurement: Self-reported response |
| **COPD treatment adherence** | Definition: Whether an individual diagnosed with COPD is currently following a treatment plan prescribed by a doctor or other healthcare professional  Functional form: Binary  Measurement: Self-reported response |
| **COPD control** | Definition: Response to the question “Has the number of acute exacerbations decreased in the past six months?”  Functional form: Binary  Measurement: Self-reported response |
| **Physical health indicators** | |
| **FEV_1_ measurement** | Definition: Forced Expiratory Volume in one second  Functional form: Continuous  Measurement: Pulmonary function test, portable spirometry |
| **Blood pressure** | Definition: Systolic and diastolic blood pressure (mmHg)  Functional form: Continuous  Measurement: Omron portable automatic blood pressure monitor |
| **Blood glucose** | Definition: Reference standard for average plasma glucose concentration over a period of time, typically reflecting the preceding 8–12 hours for fasting blood glucose and the immediate glycemic status for random blood glucose  Functional form: Continuous  Measurement: Blood glucose meter |
| **Waist circumference** | Definition: Waist circumference (cm)  Functional form: Continuous  Measurement: Soft measuring tape |
| **BMI** | Definition: Body mass index (BMI): weight divided by height squared (kg/m²)  Functional form: Continuous  Measurement: Height and weight were measured using an automated body composition analyzer. |
| **Healthcare utilization indicators** | |
| **Number of outpatient visits** | Definition: Number of outpatient visits and type of hospital visited within the past year  Functional form: Count  Measurement: Survey data |
| **Number of inpatient visits** | Definition: Number of inpatient visits and type of hospital visited within the past year  Functional form: Count  Measurement: Survey data |
| **Medical expenditure within a family over the past year** | Definition: Healthcare-related expenditures  Functional form: Continuous  Measurement: Insurance/outpatient/inpatient data |
| **Mental health indicators** | |
| **Depression symptoms** | Definition: Emotional disorders, including sadness, loss, and anger  Functional form: Continuous  Measurement: Score on Patient Health Questionnaire-9 items (PHQ-9), ^3^ which ranges from 0 to 27 with higher scores representing more severe depression symptoms |
| **Anxiety symptoms** | Definition: Unpleasant state of inner turmoil  Functional form: Continuous  Measurement: Score on General Anxiety Disorder-7 (GAD-7), ^4^ which ranges from 0 to 21 with higher scores representing more severe anxiety symptoms |
| **Warwick-Edinburgh Mental Well-being Scale (WEMWBS)** | Definition: Score reflecting overall mental health state  Functional form: Continuous  Measurement: Score on the Warwick Edinburgh Mental Well-being Scale, ^5^ which ranges from 14 to 70 with lower scores representing worse general mental health |
| **Socioeconomic profile indicators** | |
| **Productivity loss** | Definition: Productivity loss due to illness or health problems  Functional form: Continuous  Measurement: Score on the simplified Chinese version of the Work Productivity and Activity Impairment-General Health (WPAI-GH) (v2.0) questionnaire, with higher scores representing greater impairment and less productivity |
| **Care cascade indicators for hypertension and type 2 diabetes mellitus** | |
| **High blood pressure (HBP) screening** | Definition: Response to the question “Have you ever had your blood pressure measured by a doctor, nurse, or other healthcare professional?”  Functional form: Binary  Measurement: Self-reported response |
| **HBP diagnosis** | Definition: Response to the question “Have you ever been diagnosed with hypertension by a doctor?”  Functional form: Binary  Measurement: Self-reported response |
| **HBP treatment** | Definition: Response to the question “Are you currently taking any antihypertensive medication prescribed by a doctor or other healthcare professional?”  Functional form: Binary  Measurement: Self-reported response |
| **HBP control** | Definition: Blood pressure within the normal range at the end-of-year follow-up  Functional form: Binary  Measurement: Blood pressure measurement |
| **Type 2 diabetes mellitus (T2DM) screening** | Definition: Response to the question “Have you ever had your blood glucose measured by a doctor, nurse, or other healthcare professional?”  Functional form: Binary  Measurement: Self-reported response |
| **T2DM diagnosis** | Definition: Response to the question “Have you ever been diagnosed with T2DM by a doctor?”  Functional form: Binary  Measurement: Self-reported response |
| **T2DM treatment** | Definition: Response to the question “Are you currently receiving a type 2 diabetes treatment plan prescribed by a doctor or other healthcare professional?”  Functional form: Binary  Measurement: Self-reported response |
| **T2DM control** | Definition: Blood glucose within the normal range at the end-of-year follow-up  Functional form: Binary  Measurement: Blood glucose measurement |
| **Health risk behaviors** | |
| **Smoking status** | Definition: Response to the question “Do you currently smoke?”  Functional form: Binary  Measurement: Self-reported response |
| **Drinking status** | Definition: Frequency of alcohol consumption over the past three months  Functional form: Ordinal  Measurement: Self-reported response; the possible response options are: 0 = Never drink alcohol 1 = Once per month 2 = 2–3 times per month 3 = Once per week 4 = 2–3 times per week 5 = 4–6 times per week 6 = Once per day 7 = Twice per day 8 = More than twice per day 9 = Other, please specify |
| **Sugar consumption** | Definition: Frequency of consumption of sugary foods/drinks  Functional form: Ordinal  Measurement: Self-reported response; the possible response options are: 1 = almost every day/daily (6-7 days)  2 = often (4-5 days)  3 = sometimes (2-3 days)  4 = rarely or never (0-1 day) |
| **Salted vegetables consumption** | Definition: Frequency of consumption of salted vegetables  Functional form: Ordinal  Measurement: Self-reported response; the possible response options are: 1 = almost every day/daily (6-7 days)  2 = often (4-5 days)  3 = sometimes (2-3 days)  4 = rarely or never (0-1 day) |
| **Vegetable consumption** | Definition: Frequency of consumption of vegetables  Functional form: Ordinal  Measurement: Self-reported response; the possible response options are: 1 = almost every day/daily (6-7 days)  2 = often (4-5 days)  3 = sometimes (2-3 days)  4 = rarely or never (0-1 day) |
| **Physical exercise** | Definition: Frequency of physical exercise  Functional form: Ordinal  Measurement: Self-reported response; the possible response options are:  0 = No related physical activity  1 = Vigorous physical activity (e.g., lifting heavy objects, aerobic exercise, fast cycling)  2 = Moderate physical activity (e.g., lifting light objects, cycling at normal speed, playing doubles tennis; excluding walking)  3 = Light physical activity (e.g., walking) |

**Supplementary Table S2. Prospective cohort visit and data collection schedule**

| **Timepoint** | | **2024** | | | | **2025** | | | |
| --- | --- | --- | --- | --- | --- | --- | --- | --- | --- |
|  |  | **Q1** | **Q2** | **Q3** | **Q4** | **Q1** | **Q2** | **Q3** | **Q4** |
| **Sign Informed Consent** | |  | X |  |  |  |  |  |  |
| **QR Code Preliminary Screening** | |  | X |  |  |  |  |  |  |
| **Face-to-face / telephone interviews** | | | | | | | | | |
| **Demographics** | Sex, age, ethnicity, education level, marital status, employment status, occupation, income level, health insurance type |  | X |  |  |  | X |  |  |
| **Risk Factors** | Smoking, biomass fuel exposure, occupational exposure, family history (chronic respiratory diseases, including COPD, asthma, and bronchiectasis) |  | X |  |  |  | X |  |  |
| **Vaccination Inquiry** | Influenza vaccination, pneumonia vaccination |  | X |  |  |  | X |  |  |
| **Disease Information** | COPD diagnosis date |  | X |  |  |  | X |  |  |
|  | COPD severity (ventilation function, quality of life score) |  | X | X | X |  | X |  |  |
|  | COPD exacerbations within one year prior to enrollment or since the last follow-up (including symptoms, outpatient visits, ER visits, and hospitalizations) |  | X |  | X |  | X |  |  |
|  | Diabetes and hypertension management |  | X |  | X |  | X |  |  |
|  | Comorbidities |  | X |  | X |  | X |  |  |
| **Treatment** | COPD, diabetes, and hypertension treatment (care pathway treatment indicators and medication duration) |  | X |  | X |  | X |  |  |
|  | Medication and non-medication adherence (daily use, frequent use, occasional use, use only during exacerbations, or never used) |  | X | X | X |  | X |  |  |
|  | Disease-related knowledge |  |  |  |  |  | X |  |  |
| **Healthcare Resource Utilization** | Out-of-pocket/total costs for COPD, diabetes, and hypertension within one year prior to enrollment or since the last follow-up (medication, outpatient treatment, and hospitalization) |  |  |  |  |  | X |  |  |
|  | Healthcare resource utilization within one year prior to enrollment or since the last follow-up (hospital visits and hospitalization days) |  |  |  |  |  | X |  |  |
|  | Disease burden of COPD, diabetes, and hypertension within one year prior to enrollment or since the last follow-up |  |  |  |  |  | X |  |  |
| **Physical examination** | | | | | | | | | |
| **Body Measurements** | Height (m), weight (kg), BMI, waist circumference |  | X |  | X |  | X |  |  |
| **Cardiometabolic Indicators** | heart rate, blood pressure, blood glucose |  | X |  | X |  | X |  |  |
| **Spirometry** | Pre-bronchodilation test |  | X |  | X |  | X |  |  |
|  | Post-bronchodilation test |  | X |  |  |  | X |  |  |
| **Intervention** | | | | | | | | | |
| **Multi-component Interventions** | Implementation of intervention package for eligible participants |  | X |  |  |  |  |  |  |
|  | Intervention progress and adherence; re-education of non-adherent participants |  |  | X |  |  | X |  |  |
|  | Evaluation of intervention effectiveness |  |  |  |  |  | X |  |  |
| **Intention-to-treat and other analyses** | |  |  |  |  |  |  | X | X |

**Note: The proposed end date of data collection for the last participant is March 31, 2026.*

**References**

1. Luo N, Liu G, Li M, et al. Estimating an EQ-5D-5L value set for China. *Value in Health* 2017;20(4):662-69.

2. Robertson NM, Siddharthan T, Pollard SL, et al. Development and validity assessment of a chronic obstructive pulmonary disease knowledge questionnaire in low-and middle-income countries. *Annals of the American Thoracic Society* 2021;18(8):1298-305.

3. Wang W, Bian Q, Zhao Y, et al. Reliability and validity of the Chinese version of the Patient Health Questionnaire (PHQ-9) in the general population. *General hospital psychiatry* 2014;36(5):539-44.

4. Tong X, An D, McGonigal A, et al. Validation of the Generalized Anxiety Disorder-7 (GAD-7) among Chinese people with epilepsy. *Epilepsy research* 2016;120:31-36.

5. Dong A, Zhang X, Zhou H, et al. Applicability and cross-cultural validation of the Chinese version of the Warwick-Edinburgh mental well-being scale in patients with chronic heart failure. *Health and Quality of Life Outcomes* 2019;17:1-11.
