# Supplementary material for: Impact of the POPulation Medicine Multimorbidity Intervention in Xishui County (POPMIX) on people at high risk for COPD who smoke: protocol for the POPMIX-Smoking cluster randomised controlled trial
Source: BMJ Open. 2026 May 6;16(5):e112179. doi: 10.1136/bmjopen-2025-112179 (PMC13150926; doi:10.1136/bmjopen-2025-112179)
Supplement: online supplemental file 3 [file bmjopen-16-5-s003.docx]

**Version:** V4 **Participant ID:** □□□□□□□□□□

**“POPulation Medicine Multimorbidity
Intervention in Xishui County” programme
Informed Consent Form**

Dear Sir/Madam:

Hello! We sincerely invite you to participate in the research study “POPulation Medicine Multimorbidity Intervention in Xishui County programme” (POPMIX), jointly conducted by the School of Population Medicine and Public Health, Peking Union Medical College, and the National Center for Respiratory Medicine. You do not need to pay any fee to participate in this survey. This informed consent form provides information about the project to help you decide whether to take part in this study. Please read it carefully. If you have any questions, please ask the project staff. He/she will explain until you fully understand. You may discuss it with your family and friends before making your decision. Finally, please decide whether to participate in this study.

**I. Background and Purpose of the Study**

Medical research is shifting from treating disease alone to caring for health, and the focus of medicine is expanding from the health of individuals to also include the health of populations. “POPulation Medicine Multimorbidity Intervention in Xishui County” programme aims to apply Population Medicine theory, focusing on populations, to build population-level care models and indicator systems, establish a medical framework guided by Population Medicine, and work jointly with multiple sectors of society to achieve population-level health. This project contributes to China’s efforts toward the “Healthy China 2030” plan and the Sustainable Development Goals.

**II. Eligibility Criteria**

Permanent residents aged 35 years and above in Xishui County, Zunyi City, Guizhou Province.

**III. Overview of Study Procedures**

After you agree to participate in the study, the investigator will ask you questions about your personal basic information (such as name, age, sex, national ID number, and contact information), personal and household economic situation, lifestyle, disease history, and mental health status, as well as your views on certain matters. The survey will take approximately 60 minutes in total. The investigator will also conduct a physical examination, including measurement of weight, height, waist circumference, blood pressure, heart rate, and a lung function test. All records will be identified only with a study code, and all personal information will be kept strictly confidential.

After the baseline questionnaire and physical tests are completed, we will first conduct a health assessment to understand your baseline health status, including lung function, blood pressure, blood glucose, mental health, body mass index, and smoking and alcohol use. Based on the assessment results, you may be enrolled in specific health and disease care plans. These plans may include referral recommendations to higher-level hospitals, lifestyle guidance, health education, smoking cessation support, and mental health interventions.

During the study period, we will conduct one telephone follow-up at 3 months, and follow-up questionnaire visits at 6 months and 12 months to monitor changes in health status. After the study ends, if necessary, we may contact you again for further health assessments and follow-up. The follow-up time points and corresponding study activities and sample sizes are shown below.

| Time Point | Study Activities | Estimated Number of Participants |
| --- | --- | --- |
| Baseline survey | Questionnaire interview, physical examination, and intervention | Approx. 7,400 |
| 3 months after baseline (intervention group) | Telephone follow-up (questionnaire) | Approx. 3,700 |
| 6 months after baseline (intervention group) | On-site follow-up (questionnaire and physical examination) | Approx. 3,700 |
| 12 months after baseline | On-site follow-up (questionnaire and physical examination) | Approx. 7,400 |

**IV. Possible Risks and Discomforts**

During the questionnaire interview, the likelihood that questions will cause harm is extremely low. If you experience any physical or psychological discomfort at any time during the study, you have the right to refuse to answer related questions. Measurements of height, weight, and waist circumference are generally risk-free and only require standing on a measuring device or having a measuring tape placed around the waist. The risk of measuring blood pressure and heart rate is also very low; you may temporarily feel pressure from the cuff on your arm. During lung function testing, you may feel brief chest tightness or mild discomfort, but this should quickly disappear. Professionals will guide you and monitor your condition throughout the test. If you are unsure how to cooperate during the process, on-site staff will provide timely assistance. If you suffer any injury while participating in this study, we will provide necessary medical treatment.

**V. Benefits of Participation**

(1) Personal benefits: Through the questionnaire and assessments, you may better understand your health status and potential health risks. We will provide you with an on-site report of your examination results to help you understand your health status. If clinical medical intervention is needed, a physician will provide relevant recommendations. Please note that this study provides health assessment and recommendations, not medical diagnosis or treatment.

(2) Social benefits: This study will help deepen understanding of population health and provide more scientific approaches for Population Medicine and public health interventions, offering valuable evidence for the formulation and improvement of health policies in China.

**VI. Privacy and Personal Information Protection**

This study will keep your personal data, information, and privacy strictly confidential. Study data will be stored electronically on a dedicated computer and identified only by a study code, and will be used solely for scientific research. Only the principal investigator and authorized personnel may access these anonymized data to maximize information security. When necessary, government regulatory authorities, the hospital ethics committee, and other relevant researchers may review your records in accordance with regulations; however, all records will be managed in an anonymized manner (coded management and removal of all identifying information). Study results will be published in the form of statistically analyzed data, and your study records will be kept confidential as required.

**VII. Right to Refuse Participation or Withdraw**

Whether you participate in this study, or withdraw during the study, is entirely your choice. You may withdraw at any time. You will not suffer harm or punishment for refusing to participate or withdrawing, your relationship with any medical staff will not be affected, and you will not lose any benefits to which you are otherwise entitled. You may also request that we destroy the original data and personal information we have collected.

**VIII. Contacts and Contact Information**

If you have any questions regarding participant rights, or if you wish to report difficulties, dissatisfaction, or concerns encountered during participation, please contact the Public Health Subcommittee of the Ethics Review Committee of the Chinese Academy of Medical Sciences / Peking Union Medical College. Contact: Min Wang. Tel: 010-65120012.

If you would like to provide comments or suggestions about this study, you may contact study team staff member Yuhao Liu or the Scientific Research Office of Peking Union Medical College so that your feedback can be addressed in a timely manner. Study team contact: School of Population Medicine and Public Health, Chinese Academy of Medical Sciences / Peking Union Medical College, No. 31 Beijige San Tiao, Dongcheng District, Beijing. Contact: Yuhao Liu. Tel: 13031161771. Scientific Research Office contact: Scientific Research Office, Chinese Academy of Medical Sciences / Peking Union Medical College, No. 9 Dongdan San Tiao, Dongcheng District, Beijing. Contact: Qianqian Liang. Tel: 010-65104915.

**Interviewer Statement**

“I have informed the participant of the background, purpose, procedures, risks, and benefits of the ‘POPulation Medicine Multimorbidity Intervention in Xishui County’ programme, and have given him/her sufficient time to read this informed consent form, discuss with others, and have answered the questions raised. I have informed the participant that he/she may contact Yuhao Liu of the study team at any time if questions related to the survey arise, and may contact the Public Health Subcommittee of the Ethics Review Committee of the Chinese Academy of Medical Sciences / Peking Union Medical College at any time regarding his/her rights and interests, and I have provided accurate contact information. I have informed the participant that he/she may withdraw from this survey at any time. I have informed the participant that he/she will receive a copy of this informed consent form, which includes both my signature and his/her signature.”

|  |  |
| --- | --- |
| *Interviewer contact information* | *Date (YYYY/MM/DD)* |

**Participant Consent Statement**

“I have read this informed consent form and have been fully informed of the background, purpose, procedures, risks, and benefits of the ‘POPulation Medicine Multimorbidity Intervention in Xishui County’ programme’ programme. I have had sufficient time and opportunity to ask questions, and my questions have been answered satisfactorily. I voluntarily sign this informed consent form and voluntarily participate in this study. I understand that I may withdraw from this study at any time during the study period without providing any reason. I will receive a copy of this informed consent form, which includes both my signature and the interviewer’s signature.”

☐ I agree to participate in the questionnaire interview for research related to the “POPulation Medicine Multimorbidity Intervention in Xishui County” programme.

☐ I agree to participate in the physical tests for research related to the “POPulation Medicine Multimorbidity Intervention in Xishui County” programme.

☐ I agree to participate in the intervention and follow-up for research related to the “POPulation Medicine Multimorbidity Intervention in Xishui County” programme.

**Signatures:**

|  |
| --- |
| *Participant name* |

|  |  |
| --- | --- |
| *Participant signature or fingerprint  (please write in block letters)* | *Date (YYYY/MM/DD)* |

**For interviewer (please complete): Was the sample transferred in from another township?**

1. Yes. Sampled village/community: Xishui County __________ Township/Subdistrict __________ Village/Community

2. No

**Interviewer Statement**

“I have informed the participant of the background, purpose, procedures, risks, and benefits of the ‘POPulation Medicine Multimorbidity Intervention in Xishui County’ programme, and have given him/her sufficient time to read this informed consent form, discuss with others, and have answered the questions raised. I have informed the participant that he/she may contact Yuhao Liu of the study team at any time if questions related to the survey arise, and may contact the Public Health Subcommittee of the Ethics Review Committee of the Chinese Academy of Medical Sciences / Peking Union Medical College at any time regarding his/her rights and interests, and I have provided accurate contact information. I have informed the participant that he/she may withdraw from this survey at any time. I have informed the participant that he/she will receive a copy of this informed consent form, which includes both my signature and his/her signature.”

|  |  |  |
| --- | --- | --- |
| *Interviewer signature* | *Interviewer contact information* | *Date (YYYY/MM/DD)* |

**Participant Consent Statement**

“I have read this informed consent form and have been fully informed of the background, purpose, procedures, risks, and benefits of the ‘POPulation Medicine Multimorbidity Intervention in Xishui County’ programme. I have had sufficient time and opportunity to ask questions, and my questions have been answered satisfactorily. I voluntarily sign this informed consent form and voluntarily participate in this study. I understand that I may withdraw from this study at any time during the study period without providing any reason. I will receive a copy of this informed consent form, which includes both my signature and the interviewer’s signature.”

☐ I agree to participate in the questionnaire interview for research related to the “POPulation Medicine Multimorbidity Intervention in Xishui County” programme.

☐ I agree to participate in the physical tests for research related to the “POPulation Medicine Multimorbidity Intervention in Xishui County” programme.

☐ I agree to participate in the intervention and follow-up for research related to the “POPulation Medicine Multimorbidity Intervention in Xishui County” programme.

**Signatures:**

|  |
| --- |
| *Participant name* |

|  |  |
| --- | --- |
| *Participant signature or fingerprint  (please write in block letters)* | *Date (YYYY/MM/DD)* |

**For interviewer (please complete): Was the sample transferred in from another township?**

1. Yes. Sampled village/community: Xishui County __________ Township/Subdistrict __________ Village/Community

2. No
