# Supplementary material for: Impact of the POPulation Medicine Multimorbidity Intervention in Xishui County (POPMIX) on people at high risk for COPD who smoke: protocol for the POPMIX-Smoking cluster randomised controlled trial
Source: BMJ Open. 2026 May 6;16(5):e112179. doi: 10.1136/bmjopen-2025-112179 (PMC13150926; doi:10.1136/bmjopen-2025-112179)
Supplement: Supplementary file 4 [file bmjopen-2025-112179.reviewer_comments.pdf]

PEER REVIEW HISTORY

BMJ Open publishes all reviews undertaken for accepted manuscripts. Reviewers are asked to complete a checklist review form and are provided with free text boxes to elaborate on their assessment. These free text comments are reproduced below.

ARTICLE DETAILS

Title (Provisional)

Impact of the POPulation Medicine Multimorbidity Intervention in Xishui County (POPMIX) on people who smoke and are at high risk for chronic obstructive pulmonary disease: protocol for the POPMIX-Smoking cluster-randomised controlled trial

Authors

Chen , Simiao; Huang, Ke; Zheng, Zhoutao; Liu, Yuhao; Zhang, Shiyu; Chen, Wenjin; Tang, Xingyao; Cao, Zhong; Tang, Lei; Tong, xunliang; Zhao, Jinghan; He, Liu; Jiao, Lirui; Wang, Yingping; Zhao, Tianying; Luo, Yingchi; Lai, Qiande; Lyu, Xiangqin; Dou, Ruopeng; Chen, Qiushi; Bunker, A.; Vollmer, Sebastian; Geldsetzer, Pascal; Jamison, Dean; Bärnighausen, Till; Yang, Ting; Wang, Chen; Collaboration Group, POPMIX

VERSION 1 - REVIEW

**Reviewer**	**1**
**Name**	**Hepworth, Graham**
**Affiliation**	**The University of Melbourne, Statistical Consulting Centre**
**Date**	**14-Nov-2025**
**COI**	**None**

This challenging study looks worthwhile, and appears to be set up thoroughly and rigorously. The paper is written clearly and well, with few typos or ambiguities.

As a statistician, the emphasis of my review is on aspects which have a statistical component, though I comment on a few other areas which I found unclear or to be needing attention. I do not attempt to critique medical aspects outside my expertise or experience.

General comments:

The “integrated intervention” is quite complex, comprising many strands. It would surely be of interest to estimate, even roughly, the contribution of each strand to any overall effect. Please indicate how this might be done.

The towns are stratified by township population size. Stratifying is usually helpful, but needs to have a rationale, which I couldn't see in the paper. Please justify this basis for stratification.

The statistical analysis plan looks sound, though it wasn't clear how a sensitivity analysis would be conducted.

The sample size calculation looks OK, with a suitably conservative ICC of 0.05 and effect size of 3/9 = 1/3. However it is hard to see how the final numbers are derived. The numbers mentioned (7,400; 44,000; etc.) should be shown in a formula to get the final sample size of 4176 (= 2088 x 2). Such a formula needs the expected cluster size (presumably the eligible people in a town), but I can't see this anywhere, except perhaps to be derived from Table 1. Finally, where does the standard deviation of 9 come from?

Specific comments:

(page numbers refer to those printed at the bottom centre)

Page 3, line 26: "The intervention includes digital smoking cessation". Maybe "a digital smoking cessation program" would be more suitable.

Page 3, line 28 and elsewhere: "pay-for population" has a few possible meanings; it would be good to describe exactly what is meant by this here.

Page 4, line 23: Only "One limitation"? Interpreting the multi-faceted intervention might be another.

Page 4, line 25: Tidy up the punctuation.

Page 5, line 44: What exactly is the "uncertainty interval"?

Page 7, line 28: COPD patients

Page 15, line 56: Replace "and obesity" with "or obese".

Page 16, line 12: "A comprehensive incentive strategy . . . . has been introduced". It is not clear if this is part of the trial or outside it.

Page 18, line 8: "assuming an average reduction" would be better.

Page 18, line 26: I can't see how the robustness of the estimates is confirmed. Table 5 simply shows what happens.

Page 21, line 38: "issued one of the following recommendations".

This sounds like an interim analysis, but there is no description of such an analysis, just a reference in the SPIRIT checklist. Please clarify.

Page 28, line 41: Replace "are equally contributed and jointly listed" with "have equally contributed and are jointly listed".

Table 3: Some of the categorical outcomes are ordinal; it would be good to label them as such, to plan for a suitable analysis.

Page 48: Physical exercise looks more like a continuous outcome.

**Reviewer**	**2**
**Name**	**Pebley, Kinsey**
**Affiliation**	**Medical University of South Carolina, Hollings Cancer Center**
**Date**	**20-Jan-2026**
**COI**	**None**

Dear Dr. Bennet,

Thank you for the opportunity to review "Impact of POPulation Medicine Multimorbidity Intervention in Xishui County (POPMIX) on people at high risk for COPD who smoke: Protocol of the POPMIX-Smoking cluster-randomized controlled trial." I am enthusiastic about a study that considers the complexity of individuals at risk of COPD who may have comorbidities that would benefit substantially from smoking cessation. This is a large-scale study and an impressive undertaking. Overall, the paper could benefit from additional details about the intervention components, as it is unclear as to what exactly is being done, how often, and by who. Additionally, more information about recruitment procedures would be helpful, as it is unclear as to how potential participants were initially contacted to learn about and consent to participate in the study. Third, perhaps some reorganization of the methods section would help to walk readers through what is happening when. Lastly, this seems to be an adaptive trial that was not conducted as an adaptive trial, as participants in the intervention group received different intervention components depending on responses to measures or identified comorbidities. There is not a clear plan in the statistical analysis plan to tackle this. My specific comments are below:

Abstract:

1. The fact that this trial is being conducted in a rural part of China is not mentioned until the Discussion section. It may help to orient readers if this is included in the Methods.

Background and Rationale

1. The first sentence feels like an overstatement. Population health and preventive medicine have been around for a long time, and some countries and regions approach it differently, but I don't think we can call this a paradigm shift within modern medicine. The first paragraph does read a little sensational. It's not immediately clear that this paragraph fits here.

2. The second paragraph presents on deaths among middle aged Chinese men. Is there also a significant impact on women? This will help readers to understand if the planned intervention will largely target just men or men and women while also promoting equity in health research (especially since the authors mention there is an expected disproportionate increase in COPD among women in coming years).

3. The field is moving towards using person-first language. There are instances in the manuscript where the term “smoker” is used rather than “person who smokes” or similar.

4. I am enthusiastic about testing a digital intervention among rural individuals who smoke and are at high risk for COPD, and the authors’ rationale is strong. I’m not sure about the pay-for-population incentive structure because now there are multiple distinct interventions being tested simultaneously. The digital intervention rationale argues that this is a scalable way to reach populations who may not typically have access to cessation interventions, while the pay-for-population mechanism incentivizes physicians to do additional work to reduce smoking and, subsequently, smoking-related disease outcomes. These are very different. Is the point of the digital intervention not to reduce burden on physicians to treat this issue? How can you determine which intervention was responsible for any noted effects?

a. There may also be pushback from physicians with the call to “expand responsibilities” as many physicians globally are already struggling to meet patient demand within their healthcare systems. Additionally, the idea that physicians are “responsible for designing and delivering scalable, preventive strategies…” This is arguable because physicians are often not trained researchers, and thus are not responsible for designing interventions, and arguably should not be doing something like this because they lack the training to seek evidence about effectiveness. I am having a difficult time agreeing with the rationale for the pay-for-population paragraph (lines 29-41).

5. Compelling evidence for this importance of the current work is presented, but the introduction is quite lengthy and could be made more concise to save room to expand further in the methods if needed.

Methods

1. What is the rationale for including individuals who had quit smoking in the last 6 months. I recognize that they are at risk of relapse, but smoking cessation and relapse prevention may require different skills or tailoring within the intervention.

2. Page 10, line 36: “… encouraged to receive usual care.” What does this mean? Were they encouraged to talk to their doctor? What is usual care?

3. Page 10, line 40: “Stratified screening was conducted based on population characteristics.” Can you elaborate?

4. Page 14, first sentence of the "Health Education" subsection: When you say all permanent residents received health education, do you mean participants in the study?

5. I'm having a difficult time following the power calculation and sample size because of the order in which things are written. Was the sample size calculated 4,176 or 7,400? If 4,176, was the 7,400 set because of the expected attrition?

6. As I read through the methods, I am unclear about the recruitment of participants. I see the inclusion/exclusion criteria and that a list of individuals was provided from the local government, but how were individuals contacted to ask if they were interested in participating?

7. I would also be interested to know more about the ethical considerations behind this. I see this was approved by an IRB, but cold contact from a roster provided by the government seems like it would require delicate considerations.

8. Is the NicQuit intervention text-message based? How are the modules presented? Are they interactive? Additional details about this intervention would be helpful to facilitate reader understanding.

9. For the health education about smoking cessation, are providers trained to provide guidance to patients? How is this tracked? Is fidelity measured? I can appreciate the real-world implications of this study and that we cannot control everything when we attempt intervention implementation, but adherence to each of these intervention components is important data to better understand what components work and which do not.

10. For the community-based spirometry, what do you mean by "pop-up alerts?" Are these text messages? How are participants enrolled to receive these?

11. For health education related to mental health symptoms, how long is this encounter? Is it repeated? Does it depend on the severity of reported problems?

12. Page 15: Why refer to underweight and overweight/obesity as weight "abnormalities?" I think it would be highly beneficial to refer to these with their scientific terms (underweight, overweight, obesity), as weight "abnormality" reads as highly stigmatizing.

13. Additional details would be beneficial related to the length/number of encounters for the weight management intervention.

14. Perhaps I am misunderstanding, but it seems like some of the metrics examined for the pay-for-population mechanism are study-related without provider involvement. For example, the protocol as written describes participants receiving alerts to take themselves to spirometry testing, but this is a metric that providers are being examined on even though they do not appear to have involvement with this step.

15. Why administer both the FTND and HSI? These are redundant measures, with the HIS having 2 questions from the FTND.

16. The order of the methods section does make it somewhat difficult to envision what the procedures were and the flow of the study. For example, the assignment of interventions comes much later in the methods section rather than with the intervention group descriptions. Some reorganizing to tell the story may make it easier to readers to understand the flow of the study procedures.

17. The PHQ and GAD are administered, but only the WEMWBS is used to trigger a mental health intervention. What is the rationale for this? What if someone indicates suicidality on the PHQ-9?

18. What digital data capture platform is being used?

19. Page 21, “Patient and public involvement” subsection: “The results of this trial will benefit population health.” There is no way to know this until results are analyzed. Although it would be surprising, perhaps there is an iatrogenic effect of the intervention. I would highly recommend removing this statement.

20. “… we recruited as many individuals as possible to spread the awareness of multimorbidity and tobacco-related NCDs.” What does this mean?

21. Consent and withdrawal: “The study manager explained the study’s aims and detailed procedures-in the presence of a witness if required.” When would a witness be required?

22. Confidentiality subsection: “Complete confidentiality” is a strong term. It is impossible to always anticipate breaches of confidentiality, so it may be worth removing or tempering this language. Steps were taken to ensure confidentiality, but promising complete confidentiality is impossible.

23. For the secondary outcome variable of conditions that are controlled, I am not sure what this means operationally. How do you define “controlled” for these conditions? This is also a particularly short follow-up period for assessing “control” of a chronic condition. Is this repeated over time? It’s difficult to ascertain when certain things are measured within this study. I do have to jump around sections within the methods to try and piece the flow of the study together, and reorganization would be helpful to better understand what is being done when.

24. For the statistical analysis, I am assuming that the control and intervention group will be compared? This is not described in the analysis section. My concern is that not everyone received the same intervention, because their assignment to intervention components depended on responses to measures or presence of comorbidities. I’m not sure how meaningful comparisons will be made. Significantly more details are needed within this section.

Discussion

1. The first couple of paragraphs repeat what is mentioned in the Introduction and could be cut or condensed to make the manuscript more concise.

2. The authors mention that public health interventions are especially important in areas like Xishui, where healthcare access may be limited, but the interventions include having healthcare providers deliver these other interventions in order to receive the pay-for-population incentive. I'm not sure how these two ideas go together. If healthcare access is limited, how does adding additional interventions overcome the barriers to access? How does this solve issues in equity to accessing care? It seems that residents who face barriers to accessing healthcare would still have difficulty accessing these interventions. While some elements such as the digital health component serve to increase access, how would they learn about it in the first place if they aren't accessing care?

3. Page 25, line 51: The introduction of the idea of improving mental health outcomes is novel here. If that's the purpose of the intervention, it would be beneficial to list it earlier in the manuscript.

4. Page 25 (lines 56-59)-page 26 (lines 3-9): Since this is a protocol paper and no results related to the trial are known, proposing future research to expand this work seems premature. I highly recommend removing this and waiting to see if the results suggest this intervention was positive.

5. The final paragraph before the conclusion presents the idea of "proactive" work by healthcare providers, such as screening and suggests that currently providers are "passive" and wait for patients to come to them. I understand the idea, but do not understand how this would be achievable even with an incentive. Are providers to cold call people in the community to ask them to come in for services? Send out flyers? It would be great to proactively screen, but I am unclear about the implementation for such a practice and whether that would at all be feasible. Providers are already incredibly overworked in many countries and this is a significant increase in workload that seems infeasible without a clear plan.

Tables

1. With how the text is spaced in Table 2 and how things are lined up, I am having significant difficulty reading it. I cannot decipher how to interpret it. Additional lines or changing how things are spaced would be very helpful. I think things across the rows go together, but I'm not sure as it is now.

2. Table 3: Are there citations for some of these measures/questions? It's unclear if many of these come from validated scales, other studies, or were created by the study team.

VERSION 1 - AUTHOR RESPONSE

Responses to Reviewer 1

We thank Reviewer 1 for the valuable and constructive comments. We sincerely appreciate the time and expertise dedicated to reviewing our manuscript and providing insightful suggestions for improvement. We have carefully considered all of the reviewer's feedback and have revised the manuscript accordingly, which we believe has strengthened the paper substantially.

This challenging study looks worthwhile, and appears to be set up thoroughly and rigorously. The paper is written clearly and well, with few typos or ambiguities. As a statistician, the emphasis of my review is on aspects which have a statistical component, though I comment on a few other areas which I found unclear or to be needing attention. I do not attempt to critique medical aspects outside my expertise or experience.

1. *The "integrated intervention" is quite complex, comprising many strands. It would surely be of interest to estimate, even roughly, the contribution of each strand to any overall effect. Please indicate how this might be done.*

Response: Thank you for this important point. We fully agree that the intervention package contains several components and understanding the contribution of individual components is scientifically valuable. The POPMIX-Smoking intervention was designed as an integrated, population-centered model, reflecting how such programmes would be implemented in real-world primary care settings. Because the components interact dynamically, it is neither conceptually meaningful nor analytically feasible to isolate "partial" effects of individual elements. For instance, improvements in respiratory symptoms may enhance psychological well-being, while better mental health may, in turn, increase engagement with smoking cessation and treatment adherence. These bidirectional pathways make it difficult to attribute effects to a single component without misrepresenting the real-world mechanisms through which the intervention operates.

Importantly, this integrated package was developed in close consultation with local policymakers, who are primarily concerned with the overall effectiveness of the model in achieving population-level health gains, rather than the decomposition of its constituent parts. The trial is therefore powered and designed to evaluate the combined effect of the intervention as a whole.

From a scientific perspective, while we are focused on the overall intervention package, we also attempted to examine the distributional effects of the individual components. We will explore the partial effects using the Regression Discontinuity Design (RDD) method. This allows us to assess the

local average treatment effect (LATE), which captures the effects of the intervention on the target population, but we do not attempt to analyze the isolated effects of individual components.

2. *The towns are stratified by township population size. Stratifying is usually helpful, but needs to have a rationale, which I couldn't see in the paper. Please justify this basis for stratification.*

   Response: We have added an explanation for the stratification by township population size in the Trial design section. The rationale is that townships in Xishui vary in population, which could correlate with differences in healthcare resources or baseline outcome levels. Stratifying by population size before randomization ensures a balance between intervention and control arms in terms of the size of the communities, thereby controlling for potential confounding due to population-related factors.

   "Townships in Xishui were stratified based on whether their population size exceeds the average for the county to improve balance in community size-related characteristics." (Page 8)

3. *The statistical analysis plan looks sound, though it wasn't clear how a sensitivity analysis would be conducted.*

   Response: Thank you for this . We have expanded the Statistical analysis section to specify the planned sensitivity analyses. Specifically, we now specify that robustness will be assessed using alternative model specifications, including different covariance structures and covariate adjustment sets.

   "We will report both unadjusted estimates and estimates adjusted for pre-specified covariates, including age, sex, education level, marital status, baseline outcome measures, and relevant comorbid conditions." (Page 18)

4. *The sample size calculation looks OK, with a suitably conservative ICC of 0.05 and effect size of 3/9 = 1/3. However it is hard to see how the final numbers are derived. The numbers mentioned (7,400; 44,000; etc.) should be shown in a formula to get the final sample size of 4176 (= 2088 x 2). Such a formula needs the expected cluster size (presumably the eligible people in*

a town), but I can't see this anywhere, except perhaps to be derived from Table 1. Finally, where does the standard deviation of 9 come from?

Response: Thank you. We have rewritten the Sample size/Power section to (i) present the formula used for a two-arm cluster randomised trial, (ii) state the implied average cluster size (approximately 308 eligible participants per township), (iii) clarify that 4,176 is the minimum required sample size under conservative assumptions, whereas 7,400 is the maximum feasible recruitment target under the field budget and will increase precision, and (iv) explain that the assumed standard deviation (9 cigarettes/day) was informed by prior mobile-based smoking cessation trials reporting variability in cigarettes per day.[1 2] We have also clarified that, although prior studies suggest reductions of approximately 3 cigarettes/day, we powered the trial to detect a smaller, conservative between-arm difference over 12 months, given uncertainty and potential attenuation over longer follow-up.

"The sample size was determined to detect a mean reduction of three cigarettes per day in the intervention group compared to the control group, based on prior studies of digital smoking cessation interventions[1 2]. The assumed standard deviation of nine cigarettes per day was derived from the variability observed in similar populations. To account for multiple primary outcomes, we applied a Bonferroni correction, setting the two-sided significance level at 1.67% (0.05/3) to maintain the overall family-wise error rate. Power was set at 80%. Sample size calculation followed the formula for population-based stratified cluster randomised controlled trials proposed by Cresp[3]. The intracluster correlation coefficient (ICC) was assumed to be 0.05, a conservative estimate based on previous community-based trials.[4] Based on population data from the 26 townships, the expected cluster size was estimated to be 308. With 13 clusters per arm, the formula yielded a required sample size of 2,088 participants per arm, or 4,176 in total. To account for an anticipated 20% loss to follow-up, the required number of participants to recruit was inflated to 5,220. Due to budgetary and operational considerations in this pragmatic trial, we set a feasible recruitment target of 7,400 high-risk participants. Given the estimated 25% prevalence of high COPD risk among adults aged ≥35 years,[5] screening approximately 44,000 individuals was planned to identify the required number of eligible participants." (Page 15)

Specific comments:

5. *Page 3, line 26: "The intervention includes digital smoking cessation". Maybe "a digital smoking cessation program" would be more suitable.*

Response: Thank you for this suggestion. We have revised the phrasing to "a digital smoking cessation programme" for clarity.

"The intervention package integrates multiple components, including a digital smoking cessation programme, digital mental health support, community-based spirometry, tailored chronic disease management, health education, and a performance-linked "pay-for-population" scheme that aligns healthcare worker reimbursement with population health outcomes." (Page 3)

6. *Page 3, line 28 and elsewhere: "pay-for population" has a few possible meanings; it would be good to describe exactly what is meant by this here.*

Response: Thank you. We agree that the term requires a clear operational definition. We have added an explicit definition early in the manuscript (Abstract and Introduction) and expanded the Methods and Discussion to explain how pay-for-population is implemented in POPMIX-Smoking. In brief, in our study pay-for-population refers to a coverage-based incentive mechanism that links supplemental payments to population-level coverage of key services along the COPD care cascade (spirometry coverage, diagnosis rate, treatment initiation rate, and reduction in acute exacerbations), rather than to individual-level clinical outcomes.

"This protocol describes a stratified, two-arm cluster randomized controlled trial (cRCT) conducted in Xishui County, a rural area of Guizhou Province, China. A total of 26 townships were stratified by population size and randomly assigned in a 1:1 ratio to receive either a multi-component intervention or usual care. Eligible participants were individuals aged 35 years or older who smoked and were at high risk for COPD, as identified by the COPD Screening Questionnaire. The intervention package integrates multiple components, including a digital smoking cessation programme, digital mental health support, community-based spirometry, tailored chronic disease management, health education, and a performance-linked "pay-for-population" scheme that aligns healthcare worker reimbursement with population health outcomes. Primary outcomes are smoking amount and nicotine dependence, and secondary outcomes include COPD-related health outcomes, hypertension, diabetes, health risk behaviors, quality of life, healthcare utilization, and productivity loss. Follow-up occurs at three, six, and 12 months." (Page 3)

7. *Page 4, line 23: Only "One limitation"? Interpreting the multi-faceted intervention might be another.*

Response: Thank you for this thoughtful observation. We agree that the inherent complexity of the multi-faceted intervention constitutes an important limitation regarding the interpretability of its constituent effects. However, as per the journal's formatting requirement, the 'Strengths and limitations of this study' section is limited to 5 bullet points relating specifically to the methods - not the results of the study. We have therefore retained this point for detailed discussion in the "Discussion" section.

"However, there are potential challenges both to implementing the intervention and evaluating its effectiveness. First, the intervention's success depends on participant engagement, particularly among older populations who may struggle with technology or behavioral change. The digital components of the intervention, if not adequately tailored, may face resistance or low uptake, especially in rural settings. Furthermore, the study's results may be influenced by local contextual factors such as healthcare infrastructure and socioeconomic status, which could limit the generalizability of the findings beyond Xishui. Additionally, while the study's design is rigorous, it is important to acknowledge that achieving high compliance rates for both the intervention and follow-up assessments could be challenging, especially in a community setting. Addressing barriers to adherence will be crucial for ensuring that the study's findings are meaningful and reliable. Finally, the multi-faceted nature of the intervention, although reflective of real-world integrated care delivery, inherently limits our ability to isolate the individual contribution of each component to the overall treatment effect. This intervention was deliberately designed as a synergistic package in close collaboration with local policymakers, who prioritize overall effectiveness over component-level decomposition. Nevertheless, to partially address this interpretive complexity, we have prespecified exploratory analyses such as regression discontinuity designs for threshold-triggered components." (Page 23)

8. *Page 4, line 25: Tidy up the punctuation.*

Response: Thanks. We have corrected the punctuation in that sentence.

9. *Page 5, line 44: What exactly is the "uncertainty interval"?*

Response: Thanks. We have clarified this by explicitly stating that it refers to a 95% uncertainty interval.

10. *Page 7, line 28: COPD patient*

Response: Thanks. We have adjusted the phrasing to "COPD patients".

11. *Page 15, line 56: Replace "and obesity" with "or obese".*

Response: Thanks. This change has been made.

12. *Page 16, line 12: "A comprehensive incentive strategy . . . . has been introduced". It is not clear if this is part of the trial or outside it.*

Response: Thanks. We have clarified that the incentive strategy (pay-for-population) is an integral part of the intervention package implemented in the intervention townships during the trial.

13. *Page 18, line 8: "assuming an average reduction" would be better.*

Response: Thank you. We agree and have revised the wording accordingly.

14. *Page 18, line 26: I can't see how the robustness of the estimates is confirmed. Table 5 simply shows what happens.*

Response: Thank you. We agree and have revised the wording to avoid overstating what the sensitivity analysis demonstrates. We now describe Table 5 as presenting minimal detectable differences under alternative assumptions (eg, ICC values), which helps readers understand how power and precision vary with these parameters.

"Table 5 shows the minimal detectable differences (MDD) in amount of smoking among people at high risk for COPD who smoke, for 13 townships in each arm for a range of combinations of values of the intracluster correlation coefficient (ICC)." (Page 16)

15. *Page 21, line 38: "issued one of the following recommendations". This sounds like an interim analysis, but there is no description of such an analysis, just a reference in the SPIRIT checklist. Please clarify.*

Response: Thank you for the helpful observation. We have now revised the manuscript to provide a detailed and transparent description of the DSMB's activities, including the timing and content of both descriptive monitoring meetings and the prespecified interim analysis. We outline that based on this interim analysis, the DSMB will issue a recommendation to continue as planned or stop the trial.

"To ensure scientific rigour, independence and participant safety, we appointed an independent Data and Safety Monitoring Board (DSMB) comprising experts in public health, epidemiology, clinical research and respiratory disease. An external advisory committee provided additional oversight and international perspectives. The DSMB convened three meetings to review descriptive summaries from baseline and follow-up assessments in the intervention arm for process monitoring. including adherence to intervention components, implementation logistics, and data completeness and quality. A formal interim analysis was pre-specified and conducted in February 2026. At this analysis, the biostatistics team presented emerging effect estimates comparing the intervention and control arms on the primary outcomes to the DSMB and steering committee. By this time, the trial was nearing completion, as field workers confirmed that the majority of eligible participants had been contacted.

Stopping guidance was prespecified for safety, efficacy, futility and feasibility:

(1) Any death or serious adverse event was to be reported to the DSMB, which would assess relatedness to the intervention and advise whether the trial should be paused or stopped.

(2) Null findings, suggesting that the intervention had no detectable effect and indicating that the study should be terminated

(3) Statistically significant findings, indicating that the study had achieved its primary endpoint and should be concluded.

(4) If a positive trend is observed but statistical significance is not reached, the trial should continue as planned to determine the robustness of the effect. However, the study would be terminated if field workers confirmed that all eligible participants had been

contacted and no additional individuals remained to undergo follow-up assessments." (Page 19)

16. *Page 28, line 41: Replace "are equally contributed and jointly listed" with "have equally contributed and are jointly listed".*

Response: Thanks. We have fixed this grammatical issue.

17. *Table 3: Some of the categorical outcomes are ordinal; it would be good to label them as such, to plan for a suitable analysis.*

Response: Thank you. We have revised Table 3 to label ordinal outcomes explicitly (eg, ordered categories of alcohol use and sugar consumption).

18. *Page 48: Physical exercise looks more like a continuous outcome.*

Response: Thank you. We have clarified the operational definition of physical activity in Table 3. In our questionnaire it is captured as an ordered categorical variable reflecting activity level, and we will analyse it accordingly.

The revised entry in Table 3 now reads:

**Physical exercise**	Definition: Frequency of physical exercise<br>Functional form: Ordinal<br>Measurement: Self-reported response; the possible response options are:<br>0 = No related physical activity<br>1 = Vigorous physical activity (e.g., lifting heavy objects, aerobic exercise, fast cycling)<br>2 = Moderate physical activity (e.g., lifting light objects, cycling at normal speed, playing doubles tennis; excluding walking)<br>3 = Light physical activity (e.g., walking)

Responses to Reviewer 2

We thank Reviewer 2 for the valuable and constructive comments. We have carefully revised the manuscript in line with your suggestions to the best of our ability, and we believe that the changes have strengthened the overall quality of the paper.

I am enthusiastic about a study that considers the complexity of individuals at risk of COPD who may have comorbidities that would benefit substantially from smoking cessation. This is a large-scale study and an impressive undertaking. Overall, the paper could benefit from additional details about the intervention components, as it is unclear as to what exactly is being done, how often, and by who. Additionally, more information about recruitment procedures would be helpful, as it is unclear as to how potential participants were initially contacted to learn about and consent to participate in the study. Third, perhaps some reorganization of the methods section would help to walk readers through what is happening when. Lastly, this seems to be an adaptive trial that was not conducted as an adaptive trial, as participants in the intervention group received different intervention components depending on responses to measures or identified comorbidities. There is not a clear plan in the statistical analysis plan to tackle this.

Abstract

1. *The fact that this trial is being conducted in a rural part of China is not mentioned until the Discussion section. It may help to orient readers if this is included in the Methods.*

Response: Thanks. We added this in the Methods.

"This protocol describes a stratified, two-arm cluster randomized controlled trial (cRCT) conducted in Xishui County, a rural area of Guizhou Province, China." (Page 3)

Background and Rationale

2. *The first sentence feels like an overstatement. Population health and preventive medicine have been around for a long time, and some countries and regions approach it differently, but I don't think we can call this a paradigm shift within modern medicine. The first paragraph does read a little sensational. It's not immediately clear that this paragraph fits here.*

Response: Thank you. We agree and have revised the opening of the Introduction to avoid overstating novelty. In the revised Introduction, we have added text to define and contextualize the concept of "population medicine" more carefully, rather than calling it a paradigm shift.

"The growing burden of chronic diseases and persistent health inequalities have highlighted the need for healthcare approaches that extend beyond conventional, reactive, and disease-centered models.[6] Population medicine, an integrative discipline that combines clinical care with public health principles, has received increasing attention for its focus on proactive, population-level health improvement.[7] Rather than treating individual conditions in isolation, population medicine aims to identify and manage prevalent, modifiable risk factors at the population level. It emphasizes comprehensive care and the coordination of individual behaviors with collective actions to maximize long-term population health and equity. By prioritizing conditions that are widespread, preventable, and amenable to broad intervention, this approach seeks to enable more efficient resource allocation and earlier, wider public health gains." (Page 5)

3. *The second paragraph presents on deaths among middle aged Chinese men. Is there also a significant impact on women? This will help readers to understand if the planned intervention will largely target just men or men and women while also promoting equity in health research (especially since the authors mention there is an expected disproportionate increase in COPD among women in coming years).*

Response: Thank you. We have expanded that background section to include information on women. We also clarify that both men and women are included in recruitment and follow-up, the intervention enrols all eligible adults regardless of sex and that sex-stratified descriptive analyses will be explored.

"Accounting for 40% of global cigarette consumption,[8] smoking is responsible for approximately 20% of all deaths among middle-aged Chinese men and 3% among women,[9] highlighting its considerable health burden." (Page 5)

4. *The field is moving towards using person-first language. There are instances in the manuscript where the term "smoker" is used rather than "person who smokes" or similar.*

   Response: Thank you for this important reminder. We have carefully reviewed the manuscript and replaced "smoker(s)" with person-first terminology (eg, "people who smoke") wherever possible, while retaining established clinical terms (eg, "current smoker" vs "former smoker") when necessary for definitional clarity.

5. *I am enthusiastic about testing a digital intervention among rural individuals who smoke and are at high risk for COPD, and the authors' rationale is strong. I'm not sure about the pay-for-population incentive structure because now there are multiple distinct interventions being tested simultaneously. The digital intervention rationale argues that this is a scalable way to reach populations who may not typically have access to cessation interventions, while the pay-for-population mechanism incentivizes physicians to do additional work to reduce smoking and, subsequently, smoking-related disease outcomes. These are very different. Is the point of the digital intervention not to reduce burden on physicians to treat this issue? How can you determine which intervention was responsible for any noted effects?*

   Response: Thank you for this thoughtful concern. In POPMIX-Smoking, the pay-for-population incentive and the digital cessation programme are intentionally combined as complementary levers acting at different points in the care pathway rather than as competing interventions. The pay-for-population component is a provider payment reform designed to shift primary care from predominantly passive, visit-based care to proactive population health management such as systematic identification, outreach, follow-up, and accountability for high-risk people who smoke. The digital component (NicQuit) then functions as an efficiency and reach "enabler" within this proactive model: it delivers standardised, evidence-based behavioural support directly to patients at low marginal cost, thereby reducing the need for repeated face-to-face counselling time while extending support to individuals with limited access to clinic-based cessation services. Importantly, both components serve the same programme theory: expanding coverage, improving management efficiency, and ultimately advancing population health and equity. They achieve this by addressing complementary

barriers. The digital tool primarily addresses patient-level access and continuity of support, while the incentive mechanism tackles the organisational and motivational question of why and how providers should prioritise proactive prevention.

Finally, we agree that attributing observed effects to any single component is challenging in a package intervention. Because this is a pragmatic cluster trial, our primary estimand is the overall effect of offering the integrated package; we therefore cannot make definitive causal claims about the isolated contribution of each component. To partially address this inherent limitation, we have prespecified several exploratory analyses aimed at generating mechanistic insights. These include regression discontinuity designs for components triggered by a clear threshold (e.g., mental health scores)

a. *There may also be pushback from physicians with the call to "expand responsibilities" as many physicians globally are already struggling to meet patient demand within their healthcare systems. Additionally, the idea that physicians are "responsible for designing and delivering scalable, preventive strategies…" This is arguable because physicians are often not trained researchers, and thus are not responsible for designing interventions, and arguably should not be doing something like this because they lack the training to seek evidence about effectiveness. I am having a difficult time agreeing with the rationale for the pay-for-population paragraph (lines 29-41).*

Response: Thank you. We fully understand the workload pressures faced by frontline physicians and appreciate that many clinicians already work at full capacity in caring for individual patients. The idea behind the pay-for-population incentive is not to increase clinicians' daily burden, but to create space—even as little as one afternoon per week, depending on local workload flexibility—for providers to engage in population-oriented tasks that help identify unmet health needs beyond the patient encounters that occur at the bedside or in the clinic. Our experience from the field suggests that even small, scheduled time commitments can meaningfully support population medicine activities without overwhelming clinicians. We believe that enabling clinicians to combine individual patient care with a modest

amount of proactive population engagement can enhance both continuity of care and early detection of high-risk individuals, ultimately benefiting the community and supporting providers' professional fulfilment. We have revised this text to emphasise a health-system and primary-care team perspective, where incentives and enabling systems support proactive outreach as part of routine public-health and chronic-disease management functions. We also added an explicit definition of pay-for-population and clarified its purpose in supporting coverage of essential services.

"To advance population health, physicians are increasingly called upon to extend their focus beyond individual clinical care and contribute to community-level health outcomes. Achieving this broader mandate, however, requires systemic support rather than an expansion of individual responsibilities in isolation. This protocol introduces a performance-linked "pay-for-population" scheme that ties financial incentives for healthcare workers to concrete population-level indicators, including spirometry coverage, diagnosis rates, treatment initiation, and reduction in acute exacerbations. By rewarding measurable improvements in population health outcomes, the pay-for-population mechanism aims to reorient primary care toward proactive, population-level management while allowing clinicians to integrate population health activities into their existing roles in a sustainable manner." (Page 7)

6. *Compelling evidence for this importance of the current work is presented, but the introduction is quite lengthy and could be made more concise to save room to expand further in the methods if needed.*

Response: Thank you for this suggestion. We have indeed revisited the Introduction and trimmed it to improve conciseness. Some background details that were perhaps excessive or tangential have been condensed or removed.

METHODS

7. *What is the rationale for including individuals who had quit smoking in the last 6 months. I recognize that they are at risk of relapse, but smoking cessation and relapse prevention may require different skills or tailoring within the intervention.*

Response: We include people who quit within the past 6 months because relapse risk is highest during the first several months after quitting, and relapse

prevention is a core part of tobacco control. NicQuit and provider-delivered cessation support include relapse-prevention content (eg, coping strategies and trigger management). From a practical perspective, excluding early abstainers would fragment care delivery and create operational complexity in routine primary care, where smoking status is dynamic. Including this group also aligns with the population health goal of maintaining cessation and preventing relapse, not merely initiating it. To examine potential differential effects, we could further conduct subgroup analyses by smoking status.

8. *Page 10, line 36: "… encouraged to receive usual care." What does this mean? Were they encouraged to talk to their doctor? What is usual care?*

   Response: Thank you. We have revised the manuscript to clarify the definition of "usual care" for the control arm. In brief, usual care follows a traditional and largely reactive care model, in which residents seek services on their own when symptoms arise rather than through proactive population-based management. We have added this clarification in the Methods section.

   "In Xishui, usual care largely follows a routine, reactive model, in which residents seek care on their own initiative when they experience symptoms rather than through proactive population management." (Page 11)

9. *Page 10, line 40: "Stratified screening was conducted based on population characteristics." Can you elaborate?*

   Response: Thank you. We have revised this wording to clarify that "stratified screening" refers to risk stratification based on baseline assessments (eg, COPD risk, mental health symptoms, BMI, blood pressure and glucose), which determines which intervention modules a participant receives within the intervention package.

10. *Page 14, first sentence of the "Health Education" subsection: When you say all permanent residents received health education, do you mean participants in the study?*

    Response: Thank you. We have clarified that the general health-education activities are delivered at the community level in intervention townships as part of routine outreach and are not limited to trial participants.

"All permanent residents in the intervention townships receive general health education." (Page 11)

11. *I'm having a difficult time following the power calculation and sample size because of the order in which things are written. Was the sample size calculated 4,176 or 7,400? If 4,176, was the 7,400 set because of the expected attrition?*

Response: Thank you. We have rewritten the Sample size/Power section to clearly distinguish the minimum required sample size (4,176) from the budget-limited recruitment target (7,400). The minimum required sample size is based on conservative assumptions for the primary outcome under the cluster design, whereas the 7,400 figure reflects the maximum feasible enrolment based on field implementation capacity and budget. Recruiting above the minimum will improve precision and provide additional buffer against loss to follow-up.

"The sample size was determined to detect a mean reduction of three cigarettes per day in the intervention group compared to the control group, based on prior studies of digital smoking cessation interventions[1 2]. The assumed standard deviation of nine cigarettes per day was derived from the variability observed in similar populations. To account for multiple primary outcomes, we applied a Bonferroni correction, setting the two-sided significance level at 1.67% (0.05/3) to maintain the overall family-wise error rate. Power was set at 80%. Sample size calculation followed the formula for population-based stratified cluster randomised controlled trials proposed by Cresp[3]. The intracluster correlation coefficient (ICC) was assumed to be 0.05, a conservative estimate based on previous community-based trials.[4] Based on population data from the 26 townships, the expected cluster size was estimated to be 308. With 13 clusters per arm, the formula yielded a required sample size of 2,088 participants per arm, or 4,176 in total. To account for an anticipated 20% loss to follow-up, the required number of participants to recruit was inflated to 5,220. Due to budgetary and operational considerations in this pragmatic trial, we set a feasible recruitment target of 7,400 high-risk participants. Given the estimated 25% prevalence of high COPD risk among adults aged ≥35 years,[5] screening approximately 44,000 individuals was planned to identify the required number of eligible participants." (Page 16)

12. *As I read through the methods, I am unclear about the recruitment of participants. I see the inclusion/exclusion criteria and that a list of individuals was provided from the local government, but how were individuals contacted to ask if they were interested in participating?*

Response: Thank you for highlighting this gap. We have added detail in the manuscript to clarify the participant contact process. In brief, before the baseline survey, community health workers and local officials contacted potentially eligible residents identified from the government roster using WeChat, phone calls, or home visits. During initial contact, residents were provided with the study information, given the opportunity to ask questions, and invited to complete the COPD Screening Questionnaire. Those who screened positive for high COPD risk (COPD-SQ ≥ 16) were then invited to attend a community survey site—organized with the assistance of local government authorities—for a comprehensive baseline assessment, including structured questionnaires, physical examination, and spirometry. Written informed consent was obtained from all participants prior to enrolment.

"Community health workers and local officials initiated contact with potentially eligible residents using phone calls or home visits. During initial contact, residents were provided with a study information sheet, given the opportunity to ask questions, and invited to attend a community survey site for eligibility screening and baseline assessment if eligible. Informed consent was obtained from all participants prior to enrolment." (Page 8)

13. *I would also be interested to know more about the ethical considerations behind this. I see this was approved by an IRB, but cold contact from a roster provided by the government seems like it would require delicate considerations.*

Response: Thank you for raising this important point. To address the specific "cold contact" concern, our recruitment workflow deliberately avoided unfamiliar, unsolicited approaches by external researchers. Initial outreach was performed by locally embedded primary healthcare and community agents (community health workers and, where appropriate, village committee personnel), who routinely conduct community-facing public health work and often already know residents. This approach aligns with the real-world organisation of primary care and public health delivery in rural China, where village doctors are responsible for elements of the national basic public health service package (including establishing and maintaining resident health records and providing health education and chronic disease management).

We also emphasised voluntariness and autonomy in several concrete ways. First, the invitation language was neutral and non-stigmatising, avoiding disclosure of any sensitive inference about an individual's smoking status during initial contact. Second, residents were explicitly informed that participation was entirely voluntary, that they could decline without explanation, and that refusal would not affect any clinical services, family doctor services, insurance, or other administrative benefits. Third, written informed consent was obtained before enrolment and before any study procedures beyond minimal eligibility screening, and participants retained the right to withdraw at any time without reprisal.

Importantly, this active, roster-based approach was not designed simply to maximise recruitment, but to ensure equitable access to research opportunities for individuals who rarely seek care proactively or who may otherwise be unaware of available health programmes—a key ethical consideration in population health research.

Finally, this recruitment approach is not unusual in large-scale population health research in China.[10-12] Our approach builds on this precedent but advances it through a collaborative team-based model: rather than researchers directly contacting residents from a government list, we embedded recruitment within the routine functions of the existing primary care system, with coordinated involvement of local government, village health workers, and county-level hospitals. This design minimises intrusiveness, leverages established trust, and ensures that recruitment is experienced as a continuity of care rather than as unsolicited external contact.

14. *Is the NicQuit intervention text-message based? How are the modules presented? Are they interactive? Additional details about this intervention would be helpful to facilitate reader understanding.*

Response: Thank you. We have expanded the NicQuit description to clarify delivery and content. NicQuit is delivered via a WeChat-based mini-program designed as an interactive, self-guided tool for smoking cessation. The intervention modules are presented through simulated, conversational dialogues that mimic familiar chat interfaces to enhance user engagement. Each module addresses a specific therapeutic theme—such as understanding smoking triggers, developing coping strategies, or building smoke-free habits—and is delivered over approximately 10–15 minutes through a structured yet flexible interaction flow. We now describe the module schedule, interactivity, and how participants enrol and access the content.

The modules are highly interactive. Users engage with the content by responding to multiple-choice questions, selecting options that reflect their smoking urges or emotional states, typing short answers to reflection prompts, and completing action plans.

"NicQuit is a WeChat-based digital smoking cessation intervention designed for individuals who are currently smoking or have quit within the past six months. The program integrates structured, interactive modules grounded in cognitive-behavioral therapy (CBT), acceptance and commitment therapy (ACT), and other evidence-based psychological frameworks to support behavior change. Core content includes strategies for managing smoking triggers, cognitive restructuring techniques, behavioral activation exercises, and reinforcement methods to support sustained abstinence. Modules are delivered through simulated conversational dialogues, enabling participants to engage with the material in an interactive and intuitive format. The intervention is specifically targeted at individuals familiar with smartphone technology, ensuring accessibility and usability. Personalized notifications and reminders are delivered through the WeChat platform, encouraging participants to regularly engage with the cessation plan and maintain adherence." (Page 12)

15. *For the health education about smoking cessation, are providers trained to provide guidance to patients? How is this tracked? Is fidelity measured? I can appreciate the real-world implications of this study and that we cannot control everything when we attempt intervention implementation, but adherence to each of these intervention components is important data to better understand what components work and which do not.*

Response: Thank you for this important question. We agree that provider training and intervention fidelity are critical for interpreting study outcomes. All providers receive standardized training at baseline and prior to each follow-up data collection wave. The training includes a comprehensive intervention manual, on-site simulation-based practice sessions, and role-play exercises to ensure consistent delivery of the health education content. To monitor fidelity, we conduct audio recordings of health education sessions delivered in the field and routinely collect process indicators from the WeChat mini-program.

16. *For the community-based spirometry, what do you mean by "pop-up alerts?" Are these text messages? How are participants enrolled to receive these?*

Response: Thank you. The "pop-up alerts" refer to automated results displayed on the screen immediately after participants complete the COPD-SQ questionnaire, not text messages. Participants access the initial screening questionnaire by scanning a QR code using WeChat, and the results are presented directly on the completion page of the same digital form. No additional enrollment or notification mechanism is required.

17. *For health education related to mental health symptoms, how long is this encounter? Is it repeated? Does it depend on the severity of reported problems?*

Response: Thank you. The health education is delivered as brief, structured oral counseling and is provided to participants with mental health symptoms at baseline and at each follow-up visit. Each interaction follows standardized scripts to ensure consistency across participants and is not tailored based on the severity of reported symptoms. The content focuses on general mental health awareness, especially anxiety or depression, with corresponding health education for anxiety and depression respectively, and is designed to be brief and educational rather than therapeutic.

18. *Page 15: Why refer to underweight and overweight/obesity as weight "abnormalities?" I think it would be highly beneficial to refer to these with their scientific terms (underweight, overweight, obesity), as weight "abnormality" reads as highly stigmatizing.*

Response: Thank you. We agree and have revised the terminology throughout the manuscript. We now use "underweight" and "overweight/obesity" rather than "weight abnormality/abnormalities."

19. *Additional details would be beneficial related to the length/number of encounters for the weight management intervention.*

Response: We thank for this helpful comment. In response, we have expanded the description of the weight management intervention to clarify its content, frequency, and duration.

"Participants with a BMI < 18.5 (underweight) or ≥ 24.0 (overweight or obese) receive structured weight management counselling at baseline and at

each follow-up visit. Each session lasts approximately 5 to 10 minutes. During these sessions, healthcare providers use motivational interviewing techniques supported by CBT informed prompts to help participants identify weight-related barriers, reflect on the perceived advantages and disadvantages of behavioural change, and set realistic, actionable goals. Counselling includes tailored advice on diet, physical activity, and daily routines to support healthy weight gain for underweight participants and gradual, safe weight loss for those with overweight or obesity. To reinforce key messages, participants are also provided with printed educational materials to take home." (Page 13)

20. *Perhaps I am misunderstanding, but it seems like some of the metrics examined for the pay-for-population mechanism are study-related without provider involvement. For example, the protocol as written describes participants receiving alerts to take themselves to spirometry testing, but this is a metric that providers are being examined on even though they do not appear to have involvement with this step.*

Response: Thank you for this important point. We agree that performance metrics should align closely with providers' actual responsibilities. In the POPMIX-Smoking trial, township primary care teams are directly accountable for organising, delivering, and documenting spirometry services. Automated reminders function solely as a supportive tool within this provider-led outreach workflow.

This design reflects two key considerations. First, spirometry capacity has historically been limited at the primary care level in China—for instance, low availability of pulmonary function testing devices in township health centres has often necessitated referral to higher-level facilities. In this trial, we deliberately decentralise spirometry to the community by equipping township sites with portable devices and training designated operators to perform standardised testing locally. This reduces access barriers and renders spirometry coverage a meaningful, provider-influenced indicator. Second, since September 2024, COPD management has been formally integrated into China's National Basic Public Health Services, reinforcing grassroots providers' responsibilities for screening, follow-up, and service delivery—further validating the appropriateness of holding providers accountable for spirometry coverage.

Crucially, providers are not passive in this process. To achieve coverage targets—and thus qualify for performance-linked incentives—township teams actively follow up with participants beyond automated reminders. Many providers make additional phone calls, organize community-based screening sessions, and arrange transportation for those who need it. Thus, the metric reflects genuine provider effort and outreach, with automated tools playing only a facilitating role.

21. *Why administer both the FTND and HSI? These are redundant measures, with the HIS having 2 questions from the FTND.*

Response: Thank you for this helpful comment. We agree that the HSI is not an independent instrument from the FTND. The HSI is a validated two-item index derived from the FTND, based on time to first cigarette after waking and cigarettes per day. In our study, participants complete the full 6-item FTND once, and the HSI score is calculated directly from the corresponding FTND items (FTND item 1 and item 4). Therefore, there is no duplicate questioning and essentially no additional participant burden beyond administering the FTND. We report both FTND and the derived HSI because dependence is frequently reported using either metric across the smoking cessation literature and in population-based studies; presenting both improves comparability and harmonisation with prior trials and observational work. In addition, the HSI has shown strong correlation with FTND and evidence of validity as a dependence indicator in large studies.

22. *The order of the methods section does make it somewhat difficult to envision what the procedures were and the flow of the study. For example, the assignment of interventions comes much later in the methods section rather than with the intervention group descriptions. Some reorganizing to tell the story may make it easier to readers to understand the flow of the study procedures.*

Response: Thank you for this helpful suggestion. We have reorganised the Methods section to better reflect the study flow. We moved the intervention assignment procedures to an earlier spot (immediately before the intervention). This way, by the time the reader finishes the Methods, they have a chronological understanding: from how participants were identified and enrolled, to how clusters were allocated, then what intervention each arm gets, then what we measure, etc.

23. *The PHQ and GAD are administered, but only the WEMWBS is used to trigger a mental health intervention. What is the rationale for this? What if someone indicates suicidality on the PHQ-9?*

Response: Thank you for highlighting this. We chose WEMWBS as the *operational trigger* because our mental health component is intentionally framed as a population-oriented well-being promotion/early support module (low-intensity and scalable). WEMWBS was originally developed as a positively worded measure of mental well-being to support population monitoring and evaluation of mental health promotion initiatives, capturing both *feeling* and *functioning* aspects of positive mental health.[13] Consistent with the two-continua model (mental well-being and mental illness symptoms are related but distinct dimensions), individuals may experience poor well-being even if they do not meet conventional symptom "caseness" thresholds on PHQ-9 or GAD-7; these individuals may still benefit from early, low-intensity psychosocial support.[14]

Empirically, WEMWBS also shows expected, substantial inverse associations with symptom measures—e.g., in a validation study, WEMWBS correlated strongly with depressive symptoms (PHQ-9; approximately r = −0.62 to −0.67) and moderately with anxiety (GAD-7; approximately r ≈ −0.46),[15] supporting its construct validity and suitability as a population-appropriate trigger for a well-being-focused support module.

All participants receive basic empathetic communication during follow-up visits. For participants endorsing suicidality on the PHQ-9, trained staff respond with empathic listening, validate the participant's distress, offer reassurance, and—if considered necessary—verbally suggest seeking care at the county hospital's mental health services.

24. *What digital data capture platform is being used?*

Response: Thank you. We have specified the electronic data capture platform used for field data entry and validation (einMatrix).

"Study data will be collected and managed using a web-based EDC system (einMatrix). All data are entered electronically in real time and are subject to automated validation to ensure accuracy and completeness." (Page 17)

25. *Page 21, "Patient and public involvement" subsection: "The results of this trial will benefit population health." There is no way to know this*

until results are analyzed. Although it would be surprising, perhaps there is an iatrogenic effect of the intervention. I would highly recommend removing this statement.

Response: Thank you. We agree and have removed this statement.

26. *"… we recruited as many individuals as possible to spread the awareness of multimorbidity and tobacco-related NCDs." What does this mean?*

    Response: Thank you. We did not intend to suggest that we expanded trial enrollment beyond the prespecified sampling frame. Our intervention is population-based and delivered through township-level activities (e.g., on-site screening/health education) that are naturally open to community members. In practice, a small number of residents who were not part of the trial sampling list voluntarily attended and completed the on-site survey. We welcomed their participation from a public-health and service-delivery perspective, but these individuals were not enrolled as trial participants and will not be included in any primary or secondary outcome analyses. All analyses will be restricted to predefined eligible participants who were selected from the government roster, met inclusion criteria, and provided informed consent, consistent with the intention-to-treat framework for the cluster randomized trial. As suggested by the editor, we have now simplified the "Patient and public involvement" statement to read "None."

27. *Consent and withdrawal: "The study manager explained the study's aims and detailed procedures-in the presence of a witness if required." When would a witness be required?*

    Response: Thank you. We have clarified that a witness was required in cases where the participant was unable to read or write (illiterate) or had limited capacity to understand the consent form on their own.

    "A study manager explained the study aims, procedures, potential risks and benefits, and answered questions, allowing sufficient time for decision-making. A witness was present when required, specifically when a participant was unable to read the consent materials (eg, illiteracy/limited literacy) or otherwise could not review the form independently. In these cases, the consent form was read aloud, the participant indicated consent by thumbprint, and the witness co-signed to attest that the information was accurately conveyed and that consent was given voluntarily before enrolment." (Page 20)

28. *Confidentiality subsection: "Complete confidentiality" is a strong term. It is impossible to always anticipate breaches of confidentiality, so it may be worth removing or tempering this language. Steps were taken to ensure confidentiality, but promising complete confidentiality is impossible.*

Response: Thank you. We have revised this wording to avoid over-promising and to describe the concrete measures used to protect confidentiality.

"Throughout the study, all data have been handled in accordance with strict confidentiality protocols, and data collection has conformed to requirements of the national legislations on data protection. Digital data are stored in password-protected files on secure servers at the Center of the Chinese Academy of Medical Sciences, with access restricted to authorized research team members only. Any data shared with third parties for research purposes are anonymized prior to transfer. Informed consent forms, laboratory records, and other participant-related documents are securely stored during the study and will be retained in locked facilities at the Co-Principal Investigator's office premises thereafter." (Page 21)

29. *For the secondary outcome variable of conditions that are controlled, I am not sure what this means operationally. How do you define "controlled" for these conditions? This is also a particularly short follow-up period for assessing "control" of a chronic condition. Is this repeated over time? It's difficult to ascertain when certain things are measured within this study. I do have to jump around sections within the methods to try and piece the flow of the study together, and reorganization would be helpful to better understand what is being done when.*

Response: Thank you for highlighting this important issue. The number of chronic conditions controlled is defined as the count of conditions meeting prespecified control thresholds among seven objectively measured indicators: COPD, asthma, depression symptoms, anxiety symptoms, BMI, hypertension, and type 2 diabetes. For each condition, "control" is operationalised using established clinical guidelines and validated instruments (e.g., no acute exacerbation in the past six months for COPD; PHQ-9 < 10 for depression; blood pressure < 140/90 mmHg for hypertension).

Regarding follow-up duration, we agree that 12 months does not capture long-term trajectories for chronic disease. Our intention is to evaluate short- to medium-term changes in risk-factor and symptom control that are clinically meaningful and feasible to detect within a pragmatic, community-based implementation trial. For respiratory disease, we operationalise "control" using exacerbation-free status over rolling 6-month windows, which is repeatedly assessed at 6 and 12 months.

30. *For the statistical analysis, I am assuming that the control and intervention group will be compared? This is not described in the analysis section. My concern is that not everyone received the same intervention, because their assignment to intervention components depended on responses to measures or presence of comorbidities. I'm not sure how meaningful comparisons will be made. Significantly more details are needed within this section.*

Resposne: We thank the reviewer for this important comment. We agree that it is essential to clarify what is being compared and why the comparison remains valid despite individual-level variation in intervention components

First, the intervention and control groups are indeed comparable at the cluster level. Randomisation was conducted at the township level, stratified by population size, and all townships followed identical participant identification, recruitment, and enrolment procedures based on a comprehensive government roster. Within each arm, the same eligibility criteria were applied, and participants were drawn from the same underlying source population. Consequently, the distributions of baseline characteristics—including comorbidities that determine eligibility for specific intervention modules—are expected to be balanced between arms. We will formally assess this balance by presenting baseline characteristics by arm.

Second, the fact that not all participants received identical intervention components does not undermine the validity of the comparison. This trial evaluates a *care delivery strategy*, not a fixed "dose" of a single treatment. The integrated POPMIX model is implemented via a prespecified algorithm: all participants in intervention townships are offered the core package, and additional modules are triggered by baseline-identified needs (e.g., mental health symptoms, obesity, or hypertension). This needs-based tailoring is an intrinsic feature of the intervention—analogous to real-world primary care, where the services provided appropriately differ

according to patient risk profiles. Importantly, “usual care” in the control arm is also heterogeneous, reflecting usual practice. Comparing outcomes under assignment to this integrated, needs-based strategy versus assignment to usual care yields the most policy-relevant estimand: the average effect of implementing the model at the population level.

Third, the primary estimand is therefore the average effect of offering the integrated care package—including its built-in tailoring—to the target population under routine conditions. This is the quantity decision-makers need when considering whether adopting such a model would improve outcomes compared with current practice. We will not claim causal attribution to any single component from the main trial comparison.

Finally, to strengthen interpretability given this tailoring, we have prespecified several supportive analyses. We will report process indicators such as coverage and uptake of digital tools, to characterise how the intervention was actually implemented. In addition, we will explore the partial effects using the Regression Discontinuity Design (RDD) method. This allows us to assess the local average treatment effect (LATE), which captures the effects of the intervention on the target population, but we do not attempt to analyze the isolated effects of individual components.

DISCUSSION

31. *The first couple of paragraphs repeat what is mentioned in the Introduction and could be cut or condensed to make the manuscript more concise.*

Response: Thank you for this editorial suggestion. We have edited the Discussion section to remove redundant background information. In the revised Discussion opening, we either condensed or deleted sentences that were restating the problem of COPD and smoking or the general rationale that had already been detailed in the Introduction. Instead, we start the Discussion more directly with a focus on the implications or unique strengths of our study.

32. *The authors mention that public health interventions are especially important in areas like Xishui, where healthcare access may be limited, but the interventions include having healthcare providers deliver these other interventions in order to receive the pay-for-population incentive. I’m not*

sure how these two ideas go together. If healthcare access is limited, how does adding additional interventions overcome the barriers to access? How does this solve issues in equity to accessing care? It seems that residents who face barriers to accessing healthcare would still have difficulty accessing these interventions. While some elements such as the digital health component serve to increase access, how would they learn about it in the first place if they aren' t accessing care?

Response: Thank you for this very thoughtful analysis. We agree that improving access in resource-limited settings requires more than simply adding interventions to existing passive care models. Our strategy addresses healthcare access through four complementary mechanisms:

1. Physical access: bringing services to the community
The pay-for-population incentive shifts providers from facility-based, reactive care to proactive outreach. Township clinicians now conduct community-based screening and follow-up visits in villages, reducing the distance barrier. Spirometry equipment was deployed to township health centers rather than remaining at the county hospital, making diagnosis available locally. For homebound or disabled residents, transportation support is provided.

2. Digital access: reducing geographic and time constraints.
The NicQuit and EmoEase digital modules enable participants to receive cessation and mental health support remotely via mobile phone, requiring no travel. To address the reviewer' s concern about initial awareness, all eligible residents were first contacted through door-to-door or telephone outreach by village doctors or community health workers—the digital component was introduced during this direct contact, not assumed as spontaneous uptake.

3. Financial access: removing cost barriers.
All screening, spirometry, and digital interventions are provided at no cost to participants. The incentive mechanism does not shift costs to patients; rather, it funds additional provider effort to reach underserved populations.

4. Informational access: building awareness through trusted channels.
Recruitment leveraged the county' s three-tier healthcare network. Village doctors—who are trusted, long-term residents of their communities—

conducted initial awareness-building and risk screening via household visits, telephone, and local health campaigns. This approach ensures that even individuals with no prior healthcare contact are actively identified and invited into care.

33. *Page 25, line 51: The introduction of the idea of improving mental health outcomes is novel here. If that's the purpose of the intervention, it would be beneficial to list it earlier in the manuscript.*

    Response: Thank you. We have mentioned mental health support as an explicit component in both the Abstract and Introduction. In addition, we have clarified in the manuscript that mental health outcomes are included as secondary outcomes, given their established associations with smoking behavior and COPD management.

34. *Page 25 (lines 56-59)-page 26 (lines 3-9): Since this is a protocol paper and no results related to the trial are known, proposing future research to expand this work seems premature. I highly recommend removing this and waiting to see if the results suggest this intervention was positive.*

    Response: Thank you. We agree and have revised this section to avoid speculative claims about expansion. We now focus the Discussion on the rationale, implementation considerations, and how planned process and outcome data will be interpreted, without implying positive results.

35. *The final paragraph before the conclusion presents the idea of "proactive" work by healthcare providers, such as screening and suggests that currently providers are "passive" and wait for patients to come to them. I understand the idea, but do not understand how this would be achievable even with an incentive. Are providers to cold call people in the community to ask them to come in for services? Send out flyers? It would be great to proactively screen, but I am unclear about the implementation for such a practice and whether that would at all be feasible. Providers are already incredibly overworked in many countries and this is a significant increase in workload that seems infeasible without a clear plan.*

    Response: Thank you. We thank the reviewer for raising this important practical concern about the feasibility of proactive work in overstretched primary care settings. Our intervention operationalises "proactive care"

through a multi-level outreach strategy designed to minimise additional burden on individual providers while maximising community reach.

Proactive engagement occurs at several levels, each tailored to local capacity:

1. Home- and village-level outreach: Village doctors (community health workers), who already conduct routine household visits as part of China's Basic Public Health Service package, perform initial contact. They invite eligible residents to participate in screening events using phone calls, WeChat messages, or brief home visits. This leverages existing relationships and routes of communication, avoiding "cold calling" by unfamiliar researchers.
2. Community-based service points: Instead of requiring residents to travel to distant township health centres, we deploy portable spirometry equipment and trained teams to central village locations for scheduled screening sessions. This reduces the distance barrier for patients and concentrates provider effort into time-limited, high-impact events rather than continuous individual follow-up.
3. Coordinated mobilisation with local government:Township authorities assist in publicising screening dates and locations through administrative channels (e.g., village broadcast systems, community meetings), which amplifies reach without placing additional demands on clinical staff.

We fully acknowledge that proactive care could increase short-term workload. To mitigate this, we have embedded the intervention into existing routines:

1. Integration into standard workflows: Screening, follow-up, and health education activities are incorporated into the regular duties of township health centres and village clinics, rather than being added as separate, un compensated tasks. The "pay-for-population" incentive provides a tangible reward for the extra effort, making it institutionally and individually worthwhile.
2. Protected time for training and feedback: All training sessions and performance feedback meetings are scheduled during normal working hours, so providers do not have to sacrifice personal time. This also reinforces that the intervention is part of their core responsibilities, not an extracurricular burden.
3. Potential long-term workload reduction - By identifying and managing high-risk individuals early, the intervention may prevent costly acute

exacerbations and reduce future demand for urgent care. Over time, this could offset the initial investment of time and resources.

4. Ongoing qualitative evaluation: We are conducting in-depth interviews with participating providers to understand their experience of workload, identify unintended burdens, and refine implementation strategies accordingly. This real-time feedback loop will help ensure that the model remains sustainable in practice.

TABLES

36. *With how the text is spaced in Table 2 and how things are lined up, I am having significant difficulty reading it. I cannot decipher how to interpret it. Additional lines or changing how things are spaced would be very helpful. I think things across the rows go together, but I'm not sure as it is now.*
Response: Thank you. We have reformatted Table 2 to improve readability (consistent alignment, spacing, and clearer grouping of intervention components and eligibility criteria).

37. *Table 3: Are there citations for some of these measures/questions? It's unclear if many of these come from validated scales, other studies, or were created by the study team.*

Response: Thank you. We have added citations in Table 3 indicating which measures come from validated scales and prior surveys. Validated scales (e.g., FTND, PHQ-9, GAD-7, WEMWBS, EQ-5D, and the COPD Knowledge Questionnaire) are now cited with references to studies that have validated their Chinese versions.

Table 3. Primary and secondary outcomes of the cRCT

**Primary outcomes**	
**Amount of smoking**	Definition: Average number of cigarettes smoked per day<br>Functional form: Continuous<br>Measurement: Self-reported response
**Smoking dependence**	Definition: A scale that measures the degree of smoking dependence<br>Functional form: Continuous<br>Measurement: Score on the Chinese version of the Fagerström Test for Nicotine Dependence (FTND),[16 17] which ranges from 0 to 10 with higher scores representing more severe nicotine dependence; additionally measured by score on the Heaviness of Smoking Index (HSI),[18] which ranges from 0 to 6 with higher scores representing worse nicotine dependence

**Secondary outcomes**	
**Quality of life indicators**	
**Self-rated health status**	Definition: General self-assessed health status<br>Functional form: Continuous<br>Measurement: EQ-5D scale, ranging from 0 to 1 continuously; 0 represents death, and 1 represents perfect health[19]
**Number of chronic conditions controlled**	Definition: The number of conditions controlled among seven objectively measured chronic health conditions (COPD, asthma, depression symptoms, anxiety symptoms, BMI, hypertension, and type 2 diabetes)<br>Functional form: Counting data<br>Measurement: Through objective physical examination or a validated scale
**Smoking-related indicators**	
**Physical proof of cigarettes**	Definition: Response to the question "Can you show me your cigarettes?"<br>Functional form: Binary<br>Measurement: Self-reported response and visible proof of cigarette possession to study field workers
**Knowledge and awareness of COPD**	
**Self-awareness of COPD**	Definition: Response to the question "Have you ever been diagnosed with COPD?"<br>Functional form: Binary<br>Measurement: Self-reported response
**COPD knowledge**	Definition: Responses to excerpted questions of the COPD Knowledge Questionnaire; selected questions ask about information the general population should know about COPD<br>Functional form: Continuous<br>Measurement: Self-reported responses to questions; scores range from 0 to 7 with higher scores indicating a greater number of correct statements chosen by the respondent[20]
**Care cascade indicators for COPD**	
**COPD screening**	Definition: Response to the question "Have you ever had a pulmonary function test?"<br>Functional form: Binary<br>Measurement: Self-reported response
**COPD treatment adherence**	Definition: Whether an individual diagnosed with COPD is currently following a treatment plan prescribed by a doctor or other healthcare professional<br>Functional form: Binary<br>Measurement: Self-reported response
**COPD control**	Definition: Response to the question "Has the number of acute exacerbations decreased in the past six months?"<br>Functional form: Binary<br>Measurement: Self-reported response
**Physical health indicators**	
**FEV_1 measurement**	Definition: Forced Expiratory Volume in one second<br>Functional form: Continuous<br>Measurement: Pulmonary function test, portable spirometry
**Blood pressure**	Definition: Systolic and diastolic blood pressure (mmHg)<br>Functional form: Continuous<br>Measurement: Omron portable automatic blood pressure monitor
**Blood glucose**	Definition: Reference standard for average plasma glucose

	concentration over a period of time, typically reflecting the preceding 8–12 hours for fasting blood glucose and the immediate glycemic status for random blood glucose<br>Functional form: Continuous<br>Measurement: Blood glucose meter
**Waist circumference**	Definition: Waist circumference (cm)<br>Functional form: Continuous<br>Measurement: Soft measuring tape
**BMI**	Definition: Body mass index (BMI): weight divided by height squared (kg/m²)<br>Functional form: Continuous<br>Measurement: Height and weight were measured using an automated body composition analyzer.
**Healthcare utilization indicators**	
**Number of outpatient visits**	Definition: Number of outpatient visits and type of hospital visited within the past year<br>Functional form: Count<br>Measurement: Survey data
**Number of inpatient visits**	Definition: Number of inpatient visits and type of hospital visited within the past year<br>Functional form: Count<br>Measurement: Survey data
**Medical expenditure within a family over the past year**	Definition: Healthcare-related expenditures<br>Functional form: Continuous<br>Measurement: Insurance/outpatient/inpatient data
**Mental health indicators**	
**Depression symptoms**	Definition: Emotional disorders, including sadness, loss, and anger<br>Functional form: Continuous<br>Measurement: Score on Patient Health Questionnaire-9 items (PHQ-9), [21] which ranges from 0 to 27 with higher scores representing more severe depression symptoms
**Anxiety symptoms**	Definition: Unpleasant state of inner turmoil<br>Functional form: Continuous<br>Measurement: Score on General Anxiety Disorder-7 (GAD-7), [22] which ranges from 0 to 21 with higher scores representing more severe anxiety symptoms
**Warwick-Edinburgh Mental Well-being Scale (WEMWBS)**	Definition: Score reflecting overall mental health state<br>Functional form: Continuous<br>Measurement: Score on the Warwick Edinburgh Mental Well-being Scale, [23] which ranges from 14 to 70 with lower scores representing worse general mental health
**Socioeconomic profile indicators**	
**Productivity loss**	Definition: Productivity loss due to illness or health problems<br>Functional form: Continuous<br>Measurement: Score on the simplified Chinese version of the Work Productivity and Activity Impairment-General Health (WPAI-GH) (v2.0) questionnaire, with higher scores representing greater impairment and less productivity
**Care cascade indicators for hypertension and type 2 diabetes mellitus**	
**High blood pressure (HBP) screening**	Definition: Response to the question "Have you ever had your blood pressure measured by a doctor, nurse, or other healthcare professional?"

	Functional form: Binary<br>Measurement: Self-reported response
**HBP diagnosis**	Definition: Response to the question "Have you ever been diagnosed with hypertension by a doctor?"<br>Functional form: Binary<br>Measurement: Self-reported response
**HBP treatment**	Definition: Response to the question "Are you currently taking any antihypertensive medication prescribed by a doctor or other healthcare professional?"<br>Functional form: Binary<br>Measurement: Self-reported response
**HBP control**	Definition: Blood pressure within the normal range at the end-of-year follow-up<br>Functional form: Binary<br>Measurement: Blood pressure measurement
**Type 2 diabetes mellitus (T2DM) screening**	Definition: Response to the question "Have you ever had your blood glucose measured by a doctor, nurse, or other healthcare professional?"<br>Functional form: Binary<br>Measurement: Self-reported response
**T2DM diagnosis**	Definition: Response to the question "Have you ever been diagnosed with T2DM by a doctor?"<br>Functional form: Binary<br>Measurement: Self-reported response
**T2DM treatment**	Definition: Response to the question "Are you currently receiving a type 2 diabetes treatment plan prescribed by a doctor or other healthcare professional?"<br>Functional form: Binary<br>Measurement: Self-reported response
**T2DM control**	Definition: Blood glucose within the normal range at the end-of-year follow-up<br>Functional form: Binary<br>Measurement: Blood glucose measurement
**Health risk behaviors**	
**Smoking status**	Definition: Response to the question "Do you currently smoke?"<br>Functional form: Binary<br>Measurement: Self-reported response
**Drinking status**	Definition: Frequency of alcohol consumption over the past three months<br>Functional form: Ordinal<br>Measurement: Self-reported response; the possible response options are:<br>0 = Never drink alcohol<br>1 = Once per month<br>2 = 2–3 times per month<br>3 = Once per week<br>4 = 2–3 times per week<br>5 = 4–6 times per week<br>6 = Once per day<br>7 = Twice per day<br>8 = More than twice per day<br>9 = Other, please specify
**Sugar consumption**	Definition: Frequency of consumption of sugary foods/drinks

	Functional form: Ordinal<br>Measurement: Self-reported response; the possible response options are:<br>1 = almost every day/daily (6-7 days)<br>2 = often (4-5 days)<br>3 = sometimes (2-3 days)<br>4 = rarely or never (0-1 day)
**Salted vegetables consumption**	Definition: Frequency of consumption of salted vegetables<br>Functional form: Ordinal<br>Measurement: Self-reported response; the possible response options are:<br>1 = almost every day/daily (6-7 days)<br>2 = often (4-5 days)<br>3 = sometimes (2-3 days)<br>4 = rarely or never (0-1 day)
**Vegetable consumption**	Definition: Frequency of consumption of vegetables<br>Functional form: Ordinal<br>Measurement: Self-reported response; the possible response options are:<br>1 = almost every day/daily (6-7 days)<br>2 = often (4-5 days)<br>3 = sometimes (2-3 days)<br>4 = rarely or never (0-1 day)
**Physical exercise**	Definition: Frequency of physical exercise<br>Functional form: Ordinal<br>Measurement: Self-reported response; the possible response options are:<br>0 = No related physical activity<br>1 = Vigorous physical activity (e.g., lifting heavy objects, aerobic exercise, fast cycling)<br>2 = Moderate physical activity (e.g., lifting light objects, cycling at normal speed, playing doubles tennis; excluding walking)<br>3 = Light physical activity (e.g., walking)

References:

1. Liao Y, Wu Q, Kelly BC, et al. Effectiveness of a text-messaging-based smoking cessation intervention (“Happy Quit”) for smoking cessation in China: A randomized controlled trial. *PLoS medicine* 2018;15(12):e1002713.
2. Tang J, Yang J, Liu Y, et al. Efficacy of WeChat-based online smoking cessation intervention (‘WeChat WeQuit’) in China: a randomised controlled trial. *EClinicalMedicine* 2023;60
3. Crespi CM. Improved Designs for Cluster Randomized Trials. *Annu Rev Public Health* 2016;37:1-16. doi: 10.1146/annurev-publhealth-032315-021702 [published Online First: 20160118]
4. Lilholt PH, Witt Udsen F, Ehlers L, et al. Telehealthcare for patients suffering from chronic obstructive pulmonary disease: effects on health-related quality of life: results from the Danish 'TeleCare North' cluster-randomised trial. *BMJ Open* 2017;7(5):e014587. doi: 10.1136/bmjopen-2016-014587 [published Online First: 20170509]
5. Huang X, Yu H, Huang E, et al. Feasibility and performance of the chronic obstructive pulmonary disease population screener and chronic obstructive pulmonary disease screening questionnaire in a Chinese physical examination center. *Journal of Thoracic Disease* 2024;16(2):1378.
6. Naghavi M, Ong KL, Aali A, et al. Global burden of 288 causes of death and life expectancy decomposition in 204 countries and territories and 811 subnational locations, 1990–2021: a systematic analysis for the Global Burden of Disease Study 2021. *The Lancet* 2024;403(10440):2100-32.
7. !!! INVALID CITATION !!! 2
8. Yang G, Wang Y, Wu Y, et al. The road to effective tobacco control in China. *The Lancet* 2015;385(9972):1019-28.
9. !!! INVALID CITATION !!! 4-6
10. Chen Z, Chen J, Collins R, et al. China Kadoorie Biobank of 0.5 million people: survey methods, baseline characteristics and long-term follow-up. *Int J Epidemiol* 2011;40(6):1652-66.
11. Xie F, Wen S, Deng A, et al. Evaluation of a community-based integrated care model (CIE) for frail older people in rural Foshan, China: study protocol for a stepped-wedge cluster randomized controlled trial {1}. *Trials* 2023;24(1):315.
12. Ding Y, Smith HJ, Fei Y, et al. Factors influencing the provision of public health services by village doctors in Hubei and Jiangxi provinces, China. *Bull World Health Organ* 2013;91:64-69.

13. Tennant R, Hiller L, Fishwick R, et al. The Warwick-Edinburgh mental well-being scale (WEMWBS): development and UK validation. *Health and Quality of life Outcomes* 2007;5(1):63.
14. Westerhof GJ, Keyes CL. Mental illness and mental health: The two continua model across the lifespan. *Journal of adult development* 2010;17(2):110-19.
15. Smith OR, Alves DE, Knapstad M, et al. Measuring mental well-being in Norway: validation of the Warwick-Edinburgh Mental Well-being Scale (WEMWBS). *Bmc Psychiatry* 2017;17(1):182.
16. Heatherton TF, Kozlowski LT, Frecker RC, et al. The Fagerström test for nicotine dependence: a revision of the Fagerstrom Tolerance Questionnaire. *British journal of addiction* 1991;86(9):1119-27.
17. Huang CL, Lin HH, Wang HH. Psychometric evaluation of the Chinese version of the Fagerstrom Tolerance Questionnaire as a measure of cigarette dependence. *Journal of advanced nursing* 2006;55(5):596-603.
18. Heatherton TF, Kozlowski LT, Frecker RC, et al. Measuring the heaviness of smoking: using self‐reported time to the first cigarette of the day and number of cigarettes smoked per day. *British journal of addiction* 1989;84(7):791-800.
19. Luo N, Liu G, Li M, et al. Estimating an EQ-5D-5L value set for China. *Value in Health* 2017;20(4):662-69.
20. Robertson NM, Siddharthan T, Pollard SL, et al. Development and validity assessment of a chronic obstructive pulmonary disease knowledge questionnaire in low-and middle-income countries. *Annals of the American Thoracic Society* 2021;18(8):1298-305.
21. Wang W, Bian Q, Zhao Y, et al. Reliability and validity of the Chinese version of the Patient Health Questionnaire (PHQ-9) in the general population. *General hospital psychiatry* 2014;36(5):539-44.
22. Tong X, An D, McGonigal A, et al. Validation of the Generalized Anxiety Disorder-7 (GAD-7) among Chinese people with epilepsy. *Epilepsy research* 2016;120:31-36.
23. Dong A, Zhang X, Zhou H, et al. Applicability and cross-cultural validation of the Chinese version of the Warwick-Edinburgh mental well-being scale in patients with chronic heart failure. *Health and Quality of Life Outcomes* 2019;17:1-11.

VERSION 2 - REVIEW

**Reviewer**	**2**
**Name**	**Pebley, Kinsey**
**Affiliation**	**Medical University of South Carolina, Hollings Cancer Center**

Date **02-Mar-2026**

COI

Thank you for the opportunity to re-review this manuscript. It has much improved and I appreciate the considerable effort the authors made to address reviewer comments. I have a few minor points below:

Introduction:

1. “A meta-analysis covering 28 countries confirmed that COPD prevalence is significantly higher among people who currently smoke (15.4%) or formerly smoked (10.7%) compared those who never smoked (4.3%).” Pg 6- typo (missing a word)

2. Some instances of term “smoker” still present, but much improved (pg. 6, tables)

3. Introduction remains very long and repetitive. I’m not sure how the introduction was condensed, unless tracked changes are not showing for those changes.

4. Significant discussion about COPD, but none of the other behaviors of interest.

Methods

1. I believe an open label design indicates that participants are aware of their study assignment. Since participants were blinded, I believe this would qualify as a single blind study.

2. Much of the Methods is in the past tense, but the study is still ongoing.

3. I am still unsure of what suggesting seeking usual care means for the control group. Were they explicitly told to talk to their current healthcare provider? Or were they told to seek care as needed? I understand that typically individuals seek care when symptoms arise, but I am unclear as to what study staff were instructed to say.

4. Under the intervention component subsections, what does “representative” permanent resident mean?

5. In the “Outcomes” section, I think there is a typo where the HSI acronym became “HIS.”

6. Are both the sample size and power calculation subsections necessary? The information is redundant.

Discussion

1. Typo in first paragraph on page 22

2. “Future research should focus on expanding this intervention to other regions, particularly those with different healthcare contexts, to validate its effectiveness across diverse settings.” This should be qualified with something along the lines of “If the intervention is determined to be effective…”

VERSION 2 - AUTHOR RESPONSE

Thank you very much for your careful review of our paper and your valuable and constructive comments. We sincerely appreciate the time and care dedicated to re-reviewing our manuscript. We have addressed each comment (quoted in italics) as follows.

Thank you for the opportunity to re-review this manuscript. It has much improved and I appreciate the considerable effort the authors made to address reviewer comments. I have a few minor points below:

Introduction

38. *"A meta-analysis covering 28 countries confirmed that COPD prevalence is significantly higher among people who currently smoke (15.4%) or formerly smoked (10.7%) compared those who never smoked (4.3%)." Pg 6- typo (missing a word)*

Response: Thank you for catching this typographical error. We have corrected the sentence in the Introduction.

"A meta-analysis covering 28 countries confirmed that COPD prevalence is significantly higher among people who currently smoke (15.4%) or formerly smoked (10.7%) compared with those who never smoked (4.3%)." (Page 6)

39. *Some instances of term "smoker" still present, but much improved (pg. 6, tables)*

Response: Thank you for this helpful comment. We fully acknowledge that the label-based noun terms "smoker" and "smokers" were still present in the previous version of the manuscript, and have now completely removed all instances of these terms across the full text and tables, in strict alignment with person-first language principles.

40. *Introduction remains very long and repetitive. I'm not sure how the introduction was condensed, unless tracked changes are not showing for those changes.*

Response: Thank you for this important comment. We agree that the Introduction still remained too long and repetitive. We have therefore

further condensed and restructured this section. The revised Introduction has been reduced from 1,183 words to 845 words through removal of overlapping epidemiological detail, tighter presentation of the population medicine rationale, and consolidation of the previously separate paragraphs on underdiagnosis, the high-risk target population, multimorbidity, and other behavioural risks into one more concise and integrated paragraphs. These changes have made the Introduction more concise and direct, with a clearer progression from disease burden and care gaps to intervention rationale and study aim. We will also ensure that all deletions and revisions are clearly visible in the marked copy.

41. *Significant discussion about COPD, but none of the other behaviors of interest.*

Response: Thank you for this valuable observation. We agree that the Introduction previously focused too heavily on COPD and smoking, while giving insufficient attention to the broader behavioural targets included in the study. We have therefore added a concise paragraph in the Introduction to explain why co-occurring health conditions and behaviours, are also relevant to this intervention.

"Many people at high risk for COPD who smoke already have respiratory symptoms, impaired quality of life, and increased healthcare use despite lacking a formal diagnosis.[22] They also commonly experience multimorbidity, including hypertension, diabetes, and mental health disorders.[23 24] In addition, smoking often clusters with other modifiable behavioural risks, including physical inactivity, unhealthy diet, and harmful alcohol use, which contribute to COPD progression and other chronic diseases.[25] These risks are interrelated and are poorly addressed by fragmented, single-disease models of care.[26 27] " (Page 6)

METHODS

42. *I believe an open label design indicates that participants are aware of their study assignment. Since participants were blinded, I believe this would qualify as a single blind study.*

Response: Thank you for this thoughtful comment. We fully acknowledge your accurate point on the standard definitions of blinding and trial design terminology, and that our original wording lacked sufficient precision on the trial's masking status.

After careful review against the CONSORT 2025 Statement and full discussion with the trial steering committee, we have confirmed that open-label remains the most methodologically conservative, transparent, and accurate descriptor for this pragmatic cluster-randomized trial, for three core reasons:

1. No formal, systematic blinding procedures were embedded in the pre-specified trial design. While we did not explicitly communicate formal allocation labels (e.g., "intervention group" or "control group") to participants to minimize expectation bias, this was a limited procedural step, not a structured blinding protocol, and no active measures were put in place to mask the nature of care delivered to participants.
2. The multi-component, non-pharmacological, community-level design made meaningful blinding inherently unfeasible. Participants could readily distinguish the active intervention package (including digital smoking cessation support, additional spirometry assessments, tailored chronic disease management, and structured proactive follow-up) from usual care alone. Frontline providers delivering the intervention also necessarily knew group allocation to implement required study procedures.
3. Describing the trial as "single-blind" would risk overstating the degree of masking achieved. This would introduce risks of reporting bias and reader misinterpretation, which we have sought to avoid by using the most conservative, factually accurate descriptor.

We therefore retained the term "open-label", but revised the manuscript to make the masking status more precise and transparent. Specifically, we now clarify that explicit allocation labels were not emphasised to participants, but that both participants and intervention providers were aware of the care delivered. In order to mitigate concerns, we tried our best to blind statisticians and most of the investigators wherever possible, which was in align with the best practices of the open-label population health trials. We believe this wording more accurately reflects the trial design and is more consistent with the reporting of pragmatic, non-pharmacological intervention trials.

"Given the nature of the intervention, this is an open-label trial. No formal blinding procedures were implemented. Healthcare providers and research team are aware of cluster-level group allocation, and participants were aware of the care they received. To minimise expectation bias, participants are not informed of their specific group allocation, and explicit labels such as "intervention group" are deliberately avoided. Statisticians and investigators will be blinded to the group assignments wherever operationally feasible, consistent with the best practices in the open-label population health and behavioural intervention trials." (Page 9)

43. *Much of the Methods is in the past tense, but the study is still ongoing.*

Response: Thank you for this important observation. We agree that in the previous version, past tense was used too broadly in the Methods. Because the trial is still ongoing and is currently in the 12-month follow-up phase, we have revised the Methods to better reflect temporal status. Specifically, we now use present tense for stable design features and ongoing protocol procedures, retain past or present perfect tense only for activities that have already been completed (eg, study launch, recruitment, baseline assessments, and completed interim follow-up visits), and use future tense for activities that have not yet been completed (eg, completion of 12-month follow-up, database lock, and final analyses).

44. *I am still unsure of what suggesting seeking usual care means for the control group. Were they explicitly told to talk to their current healthcare provider? Or were they told to seek care as needed? I understand that typically individuals seek care when symptoms arise, but I am unclear as to what study staff were instructed to say.*

Response: Thank you for this helpful request for clarification. We agree that the phrase "encouraged to receive usual care" was vague and did not accurately describe what control-arm participants actually received. In the control arm, study staff did not provide any additional counselling, proactive follow-up, referral assistance, reminder messages, or digital support, nor were participants explicitly instructed to contact their current healthcare provider.

Rather, after completing the QR code-based COPD Screening Questionnaire, individuals with a COPD-SQ score of ≥16 were shown an automated message on the questionnaire completion page on their mobile phone screen indicating that they were at high risk for COPD. This message invited them to attend the baseline face-to-face survey and advised them to seek further medical evaluation at a hospital. Beyond this automated notification, no further study-delivered intervention was provided. We have therefore revised the manuscript to replace the previous wording with a more precise operational description of the control condition.

"Participants in the control arm received a pop-up notification on their mobile device immediately after completing the QR code-based COPD-SQ

screening. Those with high COPD risk were explicitly informed of their high-risk status, invited to complete the baseline face-to-face survey, and given a general recommendation to seek usual care at regular medical institutions independently. No further study-delivered intervention is provided. In Xishui, usual care largely follows a routine, reactive model, in which residents seek care on their own initiative rather than through proactive population management." (Page 10)

45. Under the intervention component subsections, what does "representative" permanent resident mean?

Response: Thank you for this helpful question. We are glad to provide a detailed clarification of the term "representative permanent resident".

In this study, "Permanent resident" refers to individuals aged 35 years and above who had resided in the county for at least 3 months as of the study initiation, and who planned to remain in the township during the 12-month follow-up period. All enrolled residents were sourced from the complete, government-validated roster of local permanent residents, ensuring they were the target population of the local primary health care system.

The term "**representative**" is supported by our study's population-based random sampling design. Our sampling frame covered all permanent residents aged 35 years and above (meeting the above definition) across the 26 included townships, with a total eligible source population of approximately 300,000. From this full sampling frame, we randomly selected and screened about 44,000 residents, with sampling proportionate to the eligible population size of each township. These 44,000 residents underwent an initial screening using the COPD-SQ administered via QR code. We therefore retained the term "representative" because these residents were not recruited as clinic attendees or self-selected volunteers; rather, they were drawn through a population-based random sampling process intended to reflect the age-eligible permanent resident population within the county and within each township stratum.

46. In the "Outcomes" section, I think there is a typo where the HSI acronym became "HIS."

Response: Thank you for spotting this typographical error. We have corrected the acronym throughout the manuscript.

“The FTND and HSI were assessed at baseline and are reassessed at the six- and 12-month follow-ups.” (Page 13)

47. *Are both the sample size and power calculation subsections necessary? The information is redundant.*

Response: We sincerely appreciate this perceptive comment, which has helped us optimize the clarity, conciseness, and consistency of our manuscript. We fully acknowledge that the separate "Sample size" and "Power calculations" subsections contained redundant descriptions of shared core input parameters, and we have comprehensively addressed this issue in the revised manuscript.

Originally, the two subsections were designed to sequentially present two linked but distinct components of our statistical design: the first detailed the stepwise derivation of the required and final target sample size for the trial, while the second described power verification and sensitivity analyses to confirm the robustness of our sample size estimates. However, we recognize that repeated narration of identical parameters (including the expected treatment effect, standard deviation, significance level, and intracluster correlation coefficient) across the two sections created unnecessary redundancy, as the reviewer correctly noted.

In the revised manuscript, we have merged the two subsections into a single, unified Sample Size and Power Calculation section. This merged version retains all methodologically critical information. All redundant, repetitive parameter descriptions have been removed. The revised narrative flows logically from a priori sample size derivation to verification of statistical power and estimate robustness, eliminating the highlighted redundancy while preserving the full scientific rigor and transparency of our trial design.

“The sample size was determined to detectable mean reduction of three cigarettes per day in the intervention group compared to the control group, with a standard deviation of nine cigarettes per day, consistent with estimates from prior trials of digital smoking cessation interventions in Chinese populations.[51 52] To account for multiple primary outcomes, we applied a Bonferroni correction, setting the two-sided significance level at 0.025 (0.05/2) to maintain the overall family-wise error rate, with statistical power set at 80%.

Sample size calculation followed the formula for population-based stratified cluster randomised controlled trials proposed by Crespi[53]. The intracluster correlation

coefficient (ICC) was assumed to be 0.05, a conservative estimate based on previous community-based trials.[54] Based on population data from the 26 townships, the expected cluster size was estimated to be 308. With 13 clusters per arm, the formula yielded a required sample size of 2,088 participants per arm, or 4,176 in total. To account for an anticipated 20% loss to follow-up, the required number of participants to recruit was inflated to 5,220. Due to budgetary and operational considerations in this pragmatic trial, we set a feasible recruitment target of 7,400 high-risk participants. Given the estimated 25% prevalence of high COPD risk among adults aged ≥35 years,[55] screening approximately 44,000 individuals was planned to identify the required number of eligible participants.

We conducted sensitivity analyses to calculate the minimal detectable differences (MDD) in amount of smoking across a range of plausible ICC values, with 13 clusters per arm, 80% statistical power, and a two-sided significance level of 0.025. The results of these sensitivity analyses are presented in Table 4, confirming that our final sample size is sufficient to detect clinically meaningful between-group differences across a range of realistic ICC assumptions." (Page 14)

DISCUSSION

48. Typo in first paragraph on page 22

Response: Thank you for identifying this language issue. We have carefully proofread the first paragraph of the Discussion, corrected the typo, and slightly refined the wording for clarity and readability.

"This protocol describes a cRCT designed to evaluate the effectiveness of a population medicine multimorbidity intervention for people at high risk of COPD who smoke in Xishui, China. The intervention aims to address both the physical and mental health needs of this vulnerable population by integrating multimorbidity management into routine care." (Page 20)

49. "Future research should focus on expanding this intervention to other regions, particularly those with different healthcare contexts, to validate its effectiveness across diverse settings." This should be qualified with something along the lines of "If the intervention is determined to be effective…"

Response: Thank you for this helpful suggestion. We agree that, in a protocol paper, this statement should be more appropriately qualified. We have therefore revised the sentence so that any future expansion is

explicitly conditional on the intervention first being shown to be effective.

"If the intervention is shown to be effective, future research should evaluate its transferability to other regions and healthcare contexts and assess its longer-term scalability and sustainability." (Page 23)
